# Insoluble methylene-bridged glycoluril dimers as sequestrants for dyes

**DOI:** 10.3762/bjoc.21.176

**Published:** 2025-10-29

**Authors:** Suvenika Perera, Peter Y Zavalij, Lyle Isaacs

**Affiliations:** 1 Department of Chemistry and Biochemistry, University of Maryland, College Park, Maryland 20742, United States

**Keywords:** cucurbituril, dyes, sequestrants, X-ray crystallography

## Abstract

Contamination of water bodies by micropollutants including industrial dyes is a worldwide health and environmental concern. We report the design, synthesis, and characterization of a series of methylene-bridged glycoluril dimers **G2W1**–**G2W4** that are insoluble in water and that differ in the nature of their aromatic sidewalls (**G2W4**: benzene, **G2W3**: naphthalene, **G2W1** and **G2W2**: triphenylene). We tested **G2W1**–**G2W4** along with comparator **H2** as solid-state sequestrants for a panel of five dyes (methylene blue, methylene violet, acridine orange, rhodamine 6G, and methyl violet 6B). We find that catechol-walled **H2** (OH substituents) is a superior sequestrant compared to **G2W1**–**G2W4** (OMe substituents). X-ray crystal structures for **G2W1** and **G2W3** suggest that the OMe groups fill their own cavity and thereby decrease their abilities as sequestrants. **H2** achieved a removal efficiency of 94% for methylene blue whereas **G2W1** demonstrated a 64% removal efficiency for methylene violet; both sequestration processes were largely complete within 10 minutes.

## Introduction

**Figure 1:**
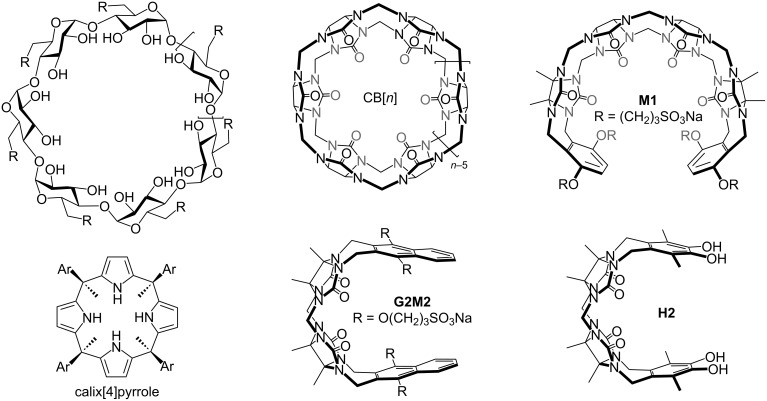
Chemical structures of selected hosts used as the basis for sequestrants.

The needs of a growing world population and the demands of modern life has resulted in the increased production of both known and new chemical substances including building materials, vitamins and minerals, cleaning products, personal care products, plastics, fertilizers, and lifesaving medicines along with deleterious substances including drugs of abuse and environmental toxins. For deleterious substances that enter the human body, in vivo antidotes are required. For example, naloxone is a well known antidote that counteracts the effects of opioid overdose by interacting with the opioid receptor, whereas the γ-cyclodextrin derivative sugammadex (Figure 1) is an in vivo sequestrant for neuromuscular blocking agents rocuronium and vecuronium and blocks their action at the nicotinic acetylcholine receptor (nAChR) by a pharmacokinetic approach [1–2]. Contamination of water bodies by the improper disposal of consumer and industrial chemicals constitutes a significant threat to the health of both humans and animals [3].

Dyes are a significant class of water pollutants which are commonly used by the textile, leather, paint, plastic, cosmetics, pharmaceuticals, and food industries [4]. It has been estimated that about 7 × 10^6^ tons of dyes (e.g., methylene blue, rhodamine B, methyl orange, Congo red, disperse violet 26, methyl red, crystal violet) are produced annually worldwide [5]. Many dyes are toxic, mutagenic, or carcinogenic and their presence, even in trace amounts, can cause issues with the kidneys, liver, brain, and central nervous system. Moreover, dyes can obstruct light penetration, affecting the photosynthetic processes in water bodies and disrupting the balance of aquatic ecosystems [5–6]. Methylene blue is a particular concern given its widespread use in the textile, paint, and food industries as well as its pharmaceutical use as a treatment for methemoglobinemia and cyanide poisoning [4]. The improvement of known and development of new methods to remove dyes from water bodies is, therefore, urgently needed.

Numerous techniques have been explored and used for the removal of dyes from water including coagulation, flocculation, adsorption, oxidation, electrolysis, biodegradation, and photocatalytic approaches [4–5]. Among these approaches, adsorption is most commonly used due to its simplicity, efficiency, low cost, and the absence of hazardous byproducts [6]. Many different adsorptive materials have been investigated including activated carbon [7–9], hybrid nanomaterials [10], metal oxide-based hybrid materials [11], metal organic frameworks [12], polymers [13], and non-conventional adsorbents [14]. Although activated carbon is widely used, its ability to capture polar compounds is limited and the regeneration process is complex and energy-intensive [15].

In work that stimulated supramolecular chemists to enter the game, Dichtel and co-workers demonstrated that β-cyclodextrin (Figure 1)-based polymers could remove organic micropollutants from water [15–16]. For example, in 2021, Sessler and co-workers reported the synthesis of a calix[4]pyrrole (Figure 1)-based porous organic polymer, which exhibits the rapid uptake of dyes from water [17]. In addition, graphene functionalized with β-cyclodextrins [18], a starch-based β-cyclodextrin polymer [19], and pillar[5]arene-based crosslinked polymers have also been investigated as sequestrants for dyes [20].

The Isaacs group has a longstanding interest in the synthesis and mechanism of formation of macrocyclic cucurbit[*n*]uril (CB[*n*]) molecular containers [21–22]. Macrocyclic CB[*n*] display ultratight binding toward hydrophobic cations in water which renders them an attractive new class of sequestrants [23–26]. For example, Buschmann and Jekel demonstrated the use of CB[6] (Figure 1) for the removal of reactive dyes from textile wastewater streams [27–29]. More recently, our group has synthesized water-soluble acyclic cucurbit[*n*]urils (e.g., **M1**, Figure 1) and demonstrated that they retain the essential molecular recognition properties of macrocyclic CB[*n*] [30]. Acyclic CB[*n*] are more easily functionalized and can flex their methylene-bridged glycoluril oligomer to accommodate guests of different size. Water-soluble acyclic CB[*n*] have been used as in vivo sequestrants for drugs of abuse, neuromuscular blockers, and anesthetics and as solubilizing agents for pharmaceuticals [31–37]. Previously, we showed that the water-soluble methylene-bridged glycoluril dimer (**G2M2**, Figure 1)-based host displayed highest affinity and selectivity for planar aromatic cations (e.g., dyes) [38]. Most recently, we synthesized a series of water-insoluble catechol-walled acyclic cucurbit[*n*]uril-type receptors (e.g., **H2**) and studied their use as sequestrants for organic micropollutants [39]. In this paper, we extend this line of inquiry toward the use of the water-insoluble glycoluril dimer-derived acyclic CB[*n*] as sequestrants for dyes.

## Results and Discussion

**Scheme 1:**
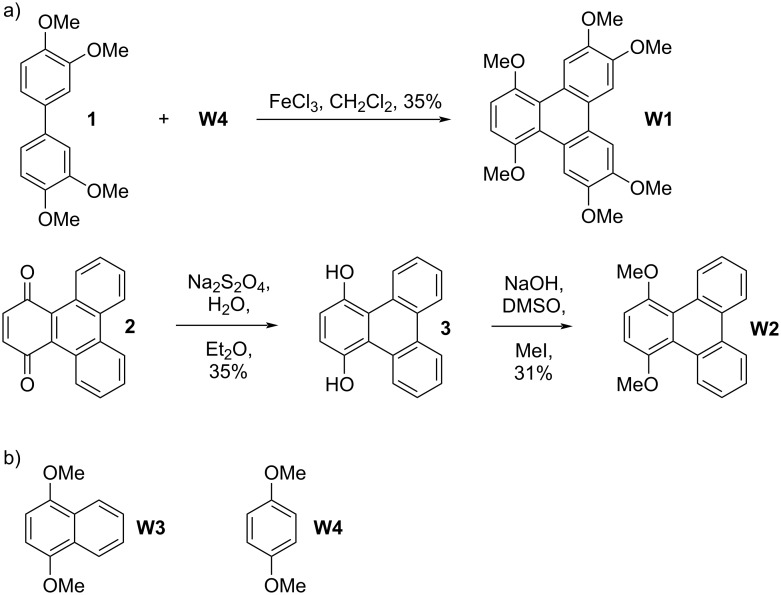
a) Synthesis of triphenylene-derived aromatic walls **W1** and **W2**, and b) structure of commercially available walls **W3** and **W4**.

**Scheme 2:**
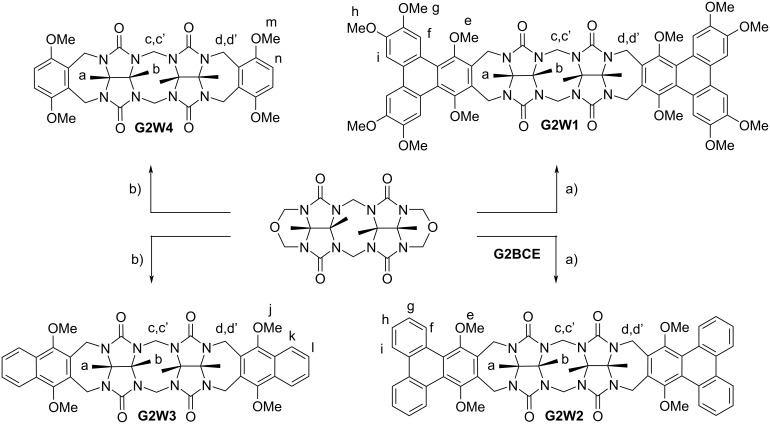
Synthesis of methylene-bridged glycoluril dimers **G2W1**–**G2W4**. Conditions: a) TFA: Ac_2_O, 95 °C, 3.5 h (**G2W1** = 28%, **G2W2** = 33%), b) TFA: Ac_2_O, 70 °C, 3.5 h (**G2W3** = 59%, **G2W4** = 62%).

**Figure 2:**
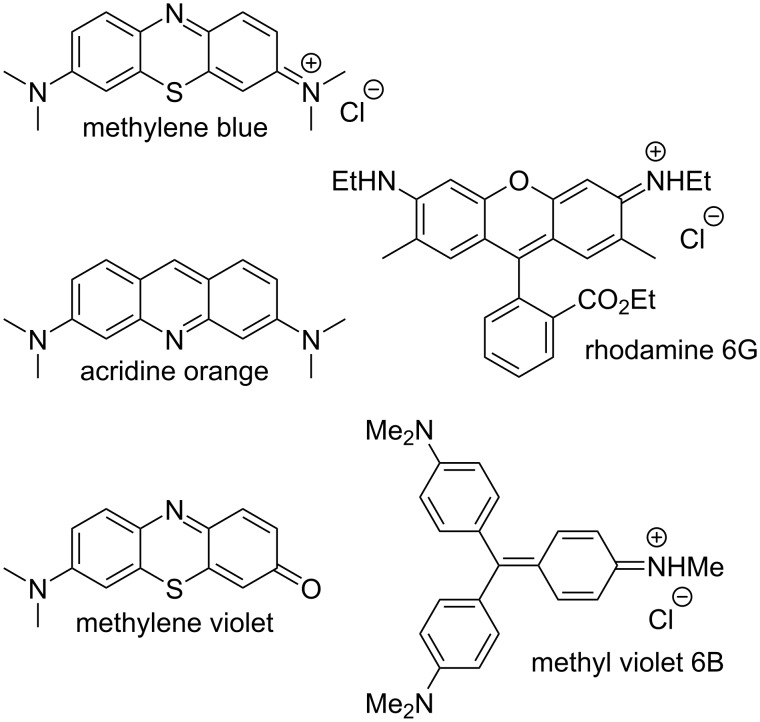
Chemical structures of dyes used in this study.

This Results and Discussion section is organized as follows. First, we present the design, synthesis, and characterization of two new aromatic walls **W1** and **W2** (Scheme 1) and four new water-insoluble acyclic CB[*n*] hosts **G2W1**–**G2W4** (Scheme 2). Next, we study the impact of different aromatic side walls on the removal of five dyes (Figure 2) from water. Subsequently, we present the X-ray crystal structures of **G2W1** and **G2W3** which helps rationalize the results from the dye sequestration experiments. Finally, we present a detailed investigation into the methylene blue removal efficiency using **H2** [39] (Figure 1) and the methylene violet removal efficiency using triphenylene-walled **G2W1**.

### Design, synthesis and characterization of **G2W1**–**G2W4**

The sequestration of large planar aromatic dyes from aqueous solution into the solid state requires water-insoluble hosts that possess complementary molecular recognition surfaces. Previously, we have studied water-soluble acyclic CB[*n*] based on methylene-bridged glycoluril monomer–tetramer and found that glycoluril tetramer-derived hosts displayed the highest binding affinity toward hydrophobic alicyclic dications due to enhanced ion–dipole interactions [40–41]. Separately, we studied glycoluril tetramer-derived acyclic CB[*n*] (e.g., **M1**) containing benzene, naphthalene, and anthracene aromatic sidewalls bearing O(CH_2_)_3_SO_3_Na water-solubilizing groups and found that the hosts with larger sidewalls displayed higher affinity toward hydrophobic alicyclic cationic guests [42–43]. Conversely, we found that the water-soluble naphthalene-walled glycoluril dimer **G2M2** (Figure 1) – with its roughly co-planar aromatic walls – is selective for planar aromatic cations as guests [38]. In order the complement the tricyclic ring system present in the panel of dyes (Figure 2), we envisioned the use of even larger aromatic walls in the form of triphenylene walls. To ensure that the targeted hosts display low aqueous solubility required for solid state sequestrants, we exchanged the O(CH_2_)_3_SO_3_Na groups for OMe groups. Accordingly, we targeted the preparation of **W1** and **W2** (Scheme 1) which contain large π-surfaces and which are activated for electrophilic aromatic substitution reactions. For the synthesis of **W1**, we initially prepared tetramethoxybiphenyl **1** (Scheme 1) according to the literature procedure involving the Suzuki coupling between 3,4-dimethoxybromobenzene and 3,4-dimethoxyphenylboronic acid [44]. Next, we performed the oxidative coupling reaction of **1** and **W4** in CH_2_Cl_2_ catalyzed by anhydrous FeCl_3_ to give **W1** in 35% yield [45]. For the synthesis of **W2**, we first oxidized triphenylene with CrO_3_ and 18-crown-6 to give triphenylene-1,4-dione (**2**) according to Echavarren’s protocol [46]. Triphenylene-1,4-dione was reduced with Na_2_S_2_O_4_ to triphenylene-1,4-diol (**3**) which was immediately treated with MeI under basic conditions (NaOH, DMSO) to give the known 1,4-dimethoxytriphenylene (**W2**) in 31% yield [47]. We also selected commercially available **W3** and **W4** to prepare comparators **G2W3** and **G2W4** to discern the effect of smaller aromatic sidewalls.

The synthesis of acyclic CB[*n*]-type receptors follows a building block approach involving the reaction of a glycoluril bis(cyclic) ether with an activated aromatic wall by a double electrophilic aromatic substitution process [30]. Scheme 2 shows the reaction of methylene-bridged glycoluril dimer **G2BCE** with aromatic walls **W1**–**W4** conducted in trifluoroacetic acid (TFA) as both solvent and acid catalyst. The new hosts **G2W1**–**G2W4** were obtained in 28, 33, 59, and 62% yield, respectively, after washing and recrystallization processes. Hosts **G2W1**–**G2W4** are insoluble in water as required for their use as solid state sequestrants. Unlike most acyclic CB[*n*]-type receptors, **G2W1**–**G2W4** are soluble in organic solvents (**G2W1** and **G2W2**: soluble in CHCl_3_, CH_2_Cl_2_, DMSO, and TFA but insoluble in methanol, acetone, acetonitrile, and hexane; **G2W3**: soluble in DMSO and TFA but insoluble in CHCl_3_, CH_2_Cl_2_, methanol, acetone, acetonitrile, and hexane; **G2W4**: soluble in CHCl_3_, CH_2_Cl_2_, acetonitrile, DMSO, and TFA but insoluble in methanol, acetone, and hexane). The new hosts **G2W1**–**G2W4** were fully characterized spectroscopically (^1^H and ^13^C NMR, IR, MS) and the data is in accord with the depicted *C*_2_*_v_*-symmetric structures.

**Figure 3:**
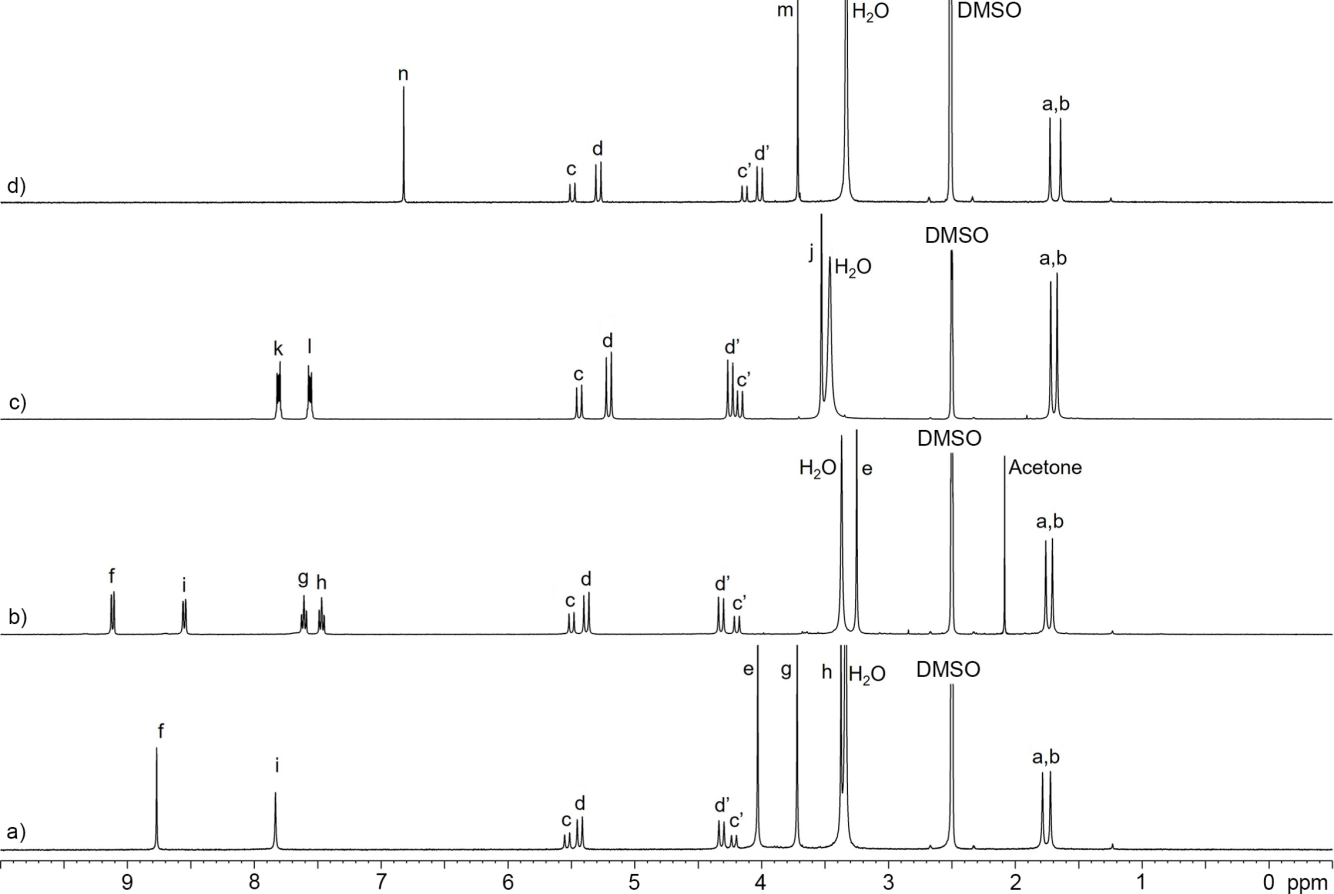
^1^H NMR spectra recorded (400 MHz, DMSO-*d_6_*, rt) for: a) **G2W1**, b) **G2W2**, c) **G2W3**, d) **G2W4**.

Figure 3 shows the ^1^H NMR spectra recorded for **G2W1**–**G2W4** in DMSO-*d*_6_ at 400 MHz. As expected, all four hosts display two singlets for the equatorial CH_3_ groups (a, b), two pairs of doublets for the diastereotopic CH_2_ groups (c,c’ and d,d’) in the expected 2:2:4:4 ratio, OCH_3_ resonances (**G2W1**: 3; **G2W2**–**G2W4**: 1), and the required aromatic resonances which are in accord with the depicted *C*_2_*_v_*-symmetric structures. Interestingly, the triphenylene bay region resonance H_f_ for **G2W1** and **G2W2** appear at 8.8 and 9.3 ppm (Figure 3a and 3b) due to the through space deshielding effect of the lone pairs on the adjacent OCH_3_ group and the neighboring aromatic ring. Similarly, the number of resonances in the ^13^C NMR recorded for **G2W1** (19 observed, 19 expected), **G2W2** (17 observed, 17 expected), **G2W3** (13 observed, 13 expected), **G2W4** (11 observed, 11 expected) are also consistent with the depicted *C*_2_*_v_*-symmetry.

### Comparison of the dye removal efficiency of hosts **H2**, **G2W1**–**G2W**4

**Figure 4:**
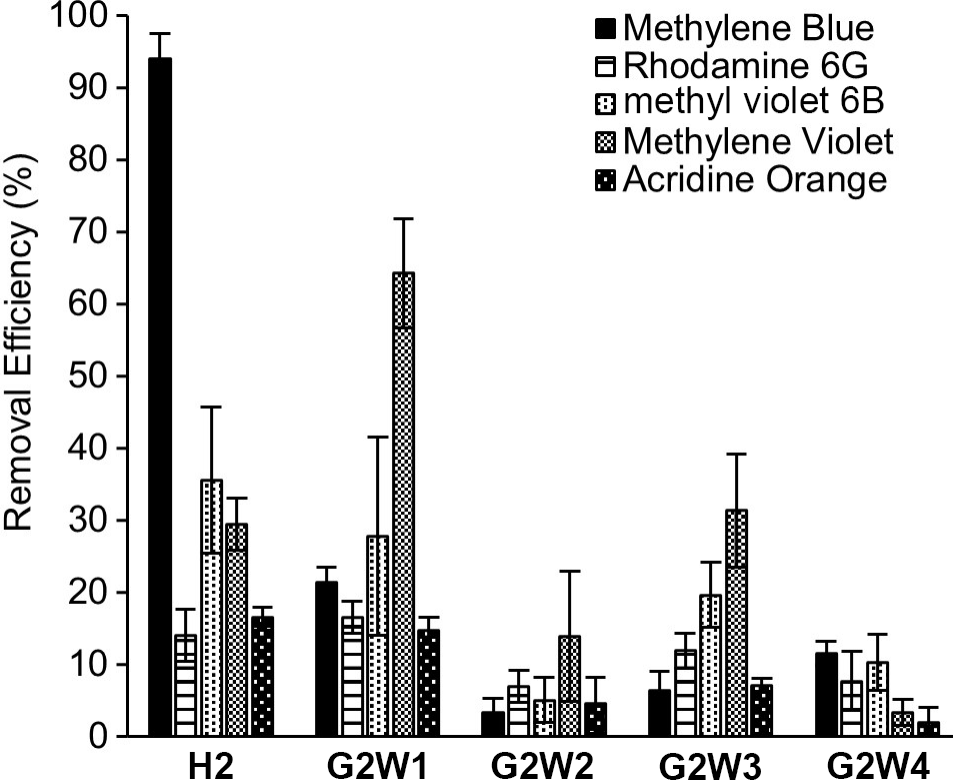
Plot of removal efficiency of dyes from water after incubating with equimolar amounts (7.2 μmol) of **H2** (5.0 mg), **G2W1** (9.0 mg), **G2W2** (7.2 mg), **G2W3** (5.7 mg), and **G2W4** (5.0 mg) for 1 hour at 25 °C as determined by UV–vis of the supernatant. Experiments were performed in triplicate (*n* = 3). Error bars represent the error propagation of uncertainty.

After firmly establishing the structures of the new water-insoluble acyclic CB[*n*] receptors **G2W1**–**G2W4**, we turned our attention to determining their efficiency as solid state sequestrants for the panel of dyes (Figure 2). We also studied the previously reported host **H2** [39], which is an isomer of **G2W4**, to potentially uncover any substituent effects (OH or OMe). For this study, we used the five dyes shown in Figure 2 (three cationic and two neutral) and employed a batch-mode experimental design. Before use, samples of **H2**, **G2W1–G2W4** were repeatedly washed with water to remove TFA (monitored by ^19^F NMR) and activated by grinding and heating overnight at 90 °C under high vacuum. Experimentally, we incubated equimolar amounts (7.2 μmol) of each host with aqueous solutions of each dye (240 μM, 1 mL) for 1 hour using a ThermoMixer™ (*T* = 25 °C, 800–1000 rpm). For the experiments with acridine orange (100 μM, 2.4 mL) and methylene violet (38 μM, 6.4 mL) lower concentrations were used due to solubility issues. Following incubation, the samples were centrifuged (11,000 rpm, 10 min for methylene blue, rhodamine 6G, methyl violet 6B; 7500 rpm, 5 min for acridine orange and methylene violet), and the supernatants were analyzed by UV–vis spectroscopy. To determine the dye concentration remaining in the aqueous solutions, appropriate calibration curves were employed (Figure S12 in Supporting Information File 1). The removal efficiency was calculated using Equation 1, where *c*_0_ is the initial dye concentration, and *c*_t_ is the dye concentration after sequestration. The results of these experiments are shown in Figure 4.


[1] $$\text{Removal efficiency (\%) }=\frac{c_{0}-c_{t}}{c_{0}}\times 100$$

Quite disappointingly, Figure 4 shows that the benzene, naphthalene, and triphenylene-walled hosts **G2W4**, **G2W3**, and **G2W2** perform poorly as solid state sequestrants across the panel of five dyes. Some glimmers of hope were seen, namely, that the removal efficiency of **H2** for methylene blue reached 94% and the removal efficiency of **G2W1** for methylene violet reached 64%. ^1^H NMR studies conducted previously with water-soluble **G2M2** established that the planar cationic dye methylene blue is encapsulated in the host’s central cavity which provides a rationale for its good removal using **H2**. Since **G2W1** fills its own cavity (vide infra), its good removal efficiency toward methylene violet likely reflects π–π stacking on the external face of **G2W1** in the solid. In contrast, the bulkier and non-planar dyes (e.g., rhodamine 6G and methyl violet 6B) are less complementary to the cavity and external faces of glycoluril dimer-based receptors. Accordingly, we decided to conduct a detailed investigation into the solid state sequestration of methylene blue by **H2** and methylene violet by **G2W1** (vide infra). We found no evidence that the increased π-surface area of the host significantly improves the adsorption behavior across the **G4W4**–**G4W2** series. Host **G2W1** – which contains a total of 12 methoxy substituents – performs significantly better than **G2W2** and displays very good removal efficiency for methylene violet. Previous researchers have shown that dye adsorption is promoted by hydroxy, carbonyl, methoxy, and aldehyde substituents which provides an explanation for the better performance of **G2W1** relative to **G2W2** [48]. Quite surprisingly, the removal efficiencies for **H2** are notably higher than **G2W4** for all five dyes studied. These differences are particularly noteworthy given that **H2** and **G2W4** are constitutional isomers and differ only in the swapping of methoxy substituents (**G2W4**) for hydroxy and methyl substituents (**H2**). Fortunately, the X-ray crystal structures of **G2W1** and **G2W3** reported below shed further light on their poor performance as solid state sequestrants.

### X-ray crystal structure of **G2W1** and **G2W3**

**Figure 5:**
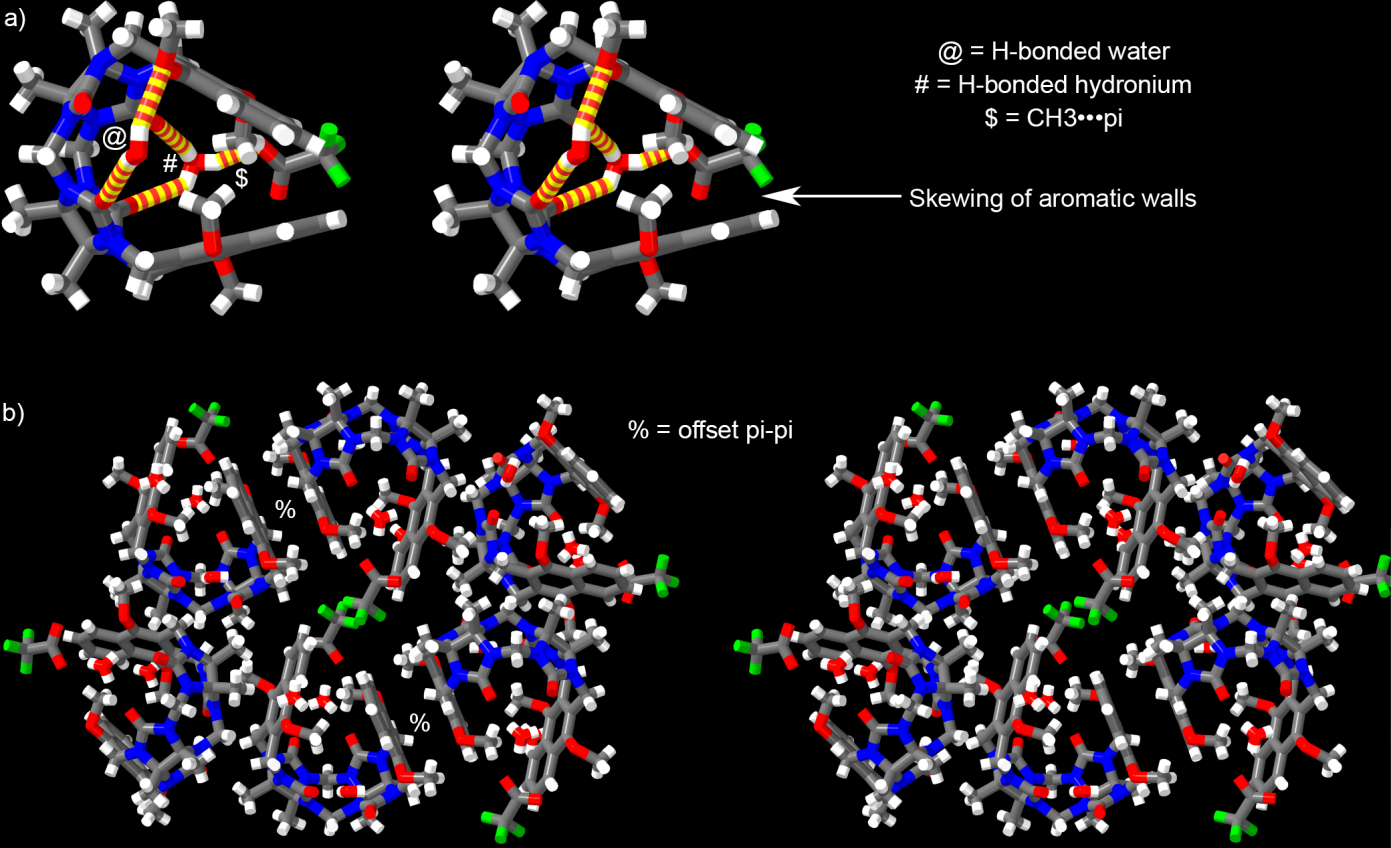
Cross-eyed stereoview of: a) one molecule of **G2W3** in the crystal, and b) the packing of **G2W3** in the crystal. Color code: C, grey; H, white; N, blue; O, red; F, green; H-bonds, yellow-red striped.

Eventually, we were able to grow single crystals of **G2W3** (CCDC 2466611) and solve their crystal structures by X-ray diffraction methods. Figure 5a shows a cross-eyed stereoview of one molecule of **G2W3** in the crystal. Crystals of **G2W3** are monoclinic with the *P*2_1_/*c* space group (*a*/Å = 10.0768(9); *b*/Å = 13.4198(11); *c*/Å = 32.411(3); α/° = 90, β/° = 98.135(3), γ/° = 90). As has been observed previously for an anthracene-walled glycoluril tetramer host [43], **G2W3** undergoes an end-to-end twist due to splaying of the aromatic sidewalls that results in an overall helical conformation that is chiral. The angle between the mean planes of the naphthalene rings amounts to 42.490°. Most relevant to the sequestration abilities of **G2W3** is the conformation of the Ar–OMe groups. The splaying of the naphthalene sidewalls positions the Ar–OCH_3_ groups directly over the opposing naphthalene sidewall and vice versa. The OCH_3_ C-atom resides 3.4925 and 3.5563 Å above the mean plane of the opposing sidewall. Solvating H_2_O and TFA are also seen in the crystal structure. At one C=O portal, an H_2_O molecule engages in H-bonding interactions with one C=O group (O···O: 2.948 Å, H···O: 2.149 Å, O–H···O angle: 163.603˚) and one OMe (O···O: 2.963 Å, H···O: 2.169 Å, O–H···O angle: 159.887°) group. The other C=O portal features interactions with H_3_O^+^ and CF_3_CO_2_^−^ groups. The H_3_O^+^ forms two H-bonds with the C=O portal (O···O: 2.740 Å, H···O: 2.117 Å, O–H···O angle: 131.315˚ and O···O: 2.688 Å, H···O: 1.866 Å, O–H···O angle: 168.483°). Figure 5b shows a cross-eyed stereoview of the packing of **G2W3** in the crystal. Two molecules of molecules of **G2W3** of opposite helicity pack in the crystal by π–π interactions (Figure 5b) between the external faces of one of the naphthalene sidewalls as seen frequently for glycoluril-derived molecular clips [49]. The distance between the mean planes of these offset stacked naphthalene rings is 3.4562 Å. The other naphthalene sidewall does not engage in π–π interactions and instead interacts with the convex face of a glycoluril unit on a separate dimeric unit of **G2W3**. The self-filling of the cavity of **G2W3** with its OCH_3_ substituents – which is not possible for **H2** with its OH and CH_3_ substituents – provides a compelling explanation for the superior performance of **H2** over **G2W3** and **G2W4**.

**Figure 6:**
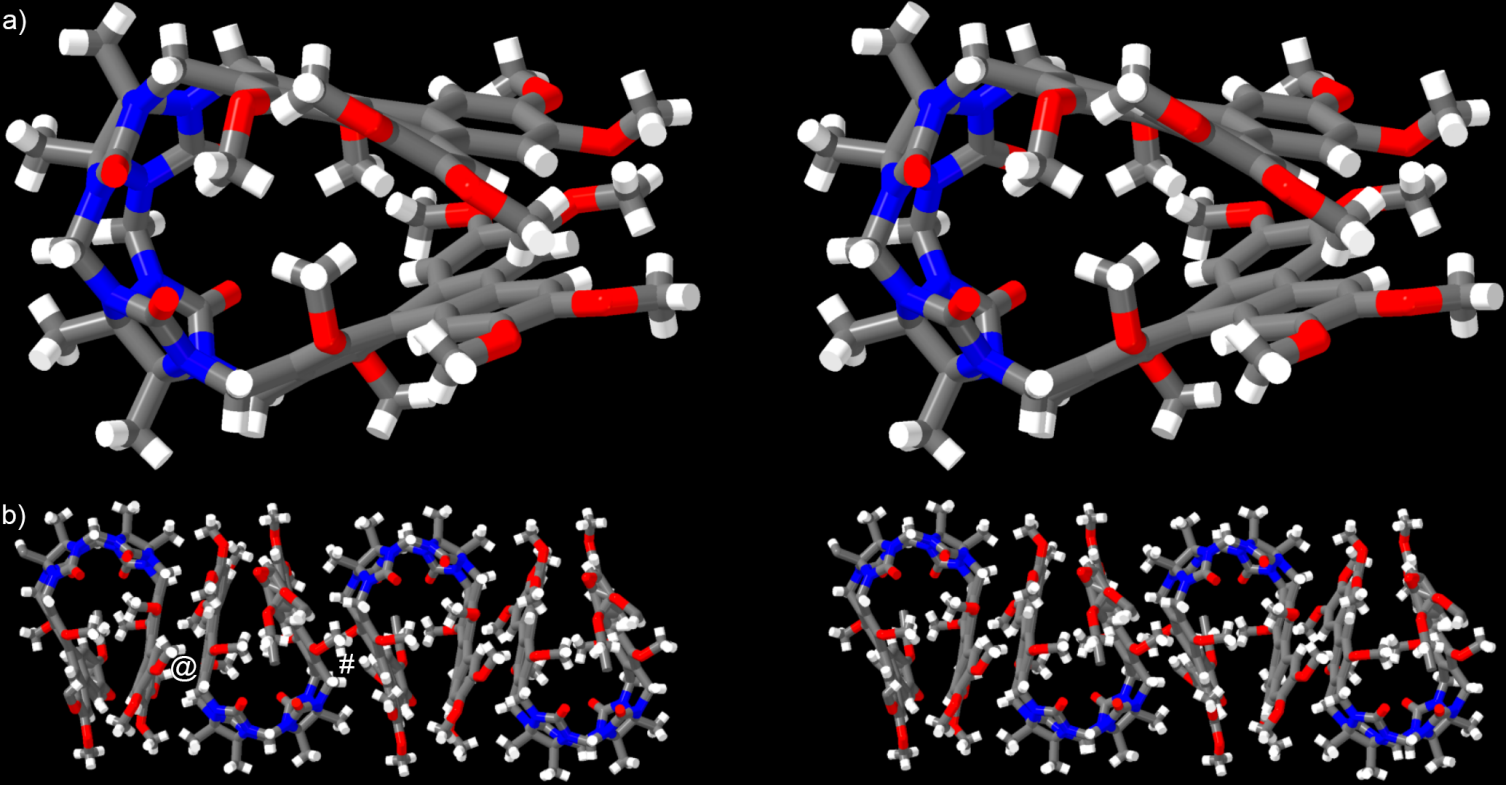
Cross-eyed stereoview of: a) a molecule of **G2W1** in the crystal, b) the packing of **G2W1** along the xz-diagonal in the crystal. Color code: C, grey; H, white; N, blue; O, red.

We were also fortunate to obtain single crystals of **G2W1** (CCDC 2466610) and solve the structure by X-ray diffraction measurements. Crystals of **G2W1** are triclinic with the *P−*1 space group (*a*/Å = 15.414(4); *b*/Å = 16.050(5); *c*/Å = 18.165(5); α/° = 64.669(7), β/° = 69.128(6), γ/° = 76.782(7)). Figure 6a shows a cross-eyed stereoview of **G2W1** in the asymmetric unit of the crystal. Similar to that observed for **G2W3**, the **G2W1** molecules undergo a splaying of their triphenylene walls. This splayed geometry allows one inwardly turned OCH_3_ group to point toward the face of the opposing triphenylene sidewall. The OCH_3_ C-atoms reside 3.7134 Å and 3.7736 Å from the mean planes of the *o*-xylylene unit of the opposing sidewall. The angle between the mean planes of the triphenylene walls is 30.856°. Another notable feature of the structure of **G2W1** is the distinctly non-planar triphenylene walls. This non-planarity is likely tied to the constraints of the preferred inward orientation of the OCH_3_ groups, but also to the need to alleviate steric interactions between Ar–H_f_ and OCH_3_ substituents in the bay region of the triphenylene ring system closest to the glycoluril dimer backbone. Both the inwardly turned OCH_3_ group which partially fills its own cavity and the distinctly non-planar triphenylene sidewalls which disrupt π–π interactions with guests allow us to rationalize the relatively poor abilities of **G2W1** as a solid state sequestrant. Figure 6b shows a cross-eyed stereoview of the packing of **G2W1** along the *xz*-diagonal via interactions between the convex surface of the triphenylene sidewalls. Molecules of **G2W1** form dimers which then further associate to form tape-like assemblies. The mean planes of the triphenylene walls within the initial dimer are co-planar with each other and the distance between the mean planes of the triphenylene sidewalls is 3.4975 Å (marked with @) which is somewhat longer than the commonly accepted π-stacking distance of 3.4 Å. The mean planes of the external triphenylene walls between the dimers are co-planar with each other and the distance between the mean planes of the external triphenylene sidewalls between the dimers is 3.5162 Å (marked with #). It should be noted that these external triphenylene walls are significantly offset with respect to each other and do not appear to engage in direct π–π interactions with each other. Overall, the X-ray crystal structures of **G2W1** and **G2W3** demonstrate that the incorporated OMe groups are deleterious for their function as solid state sequestrants because they serve to: 1) fill their own cavity, and 2) promote the non-planarity of the triphenylene sidewalls, both of which reduce their host–guest recognition abilities.

### Detailed studies of methylene blue removal by **H2** and methylene violet removal by **G2W1**

**Figure 7:**
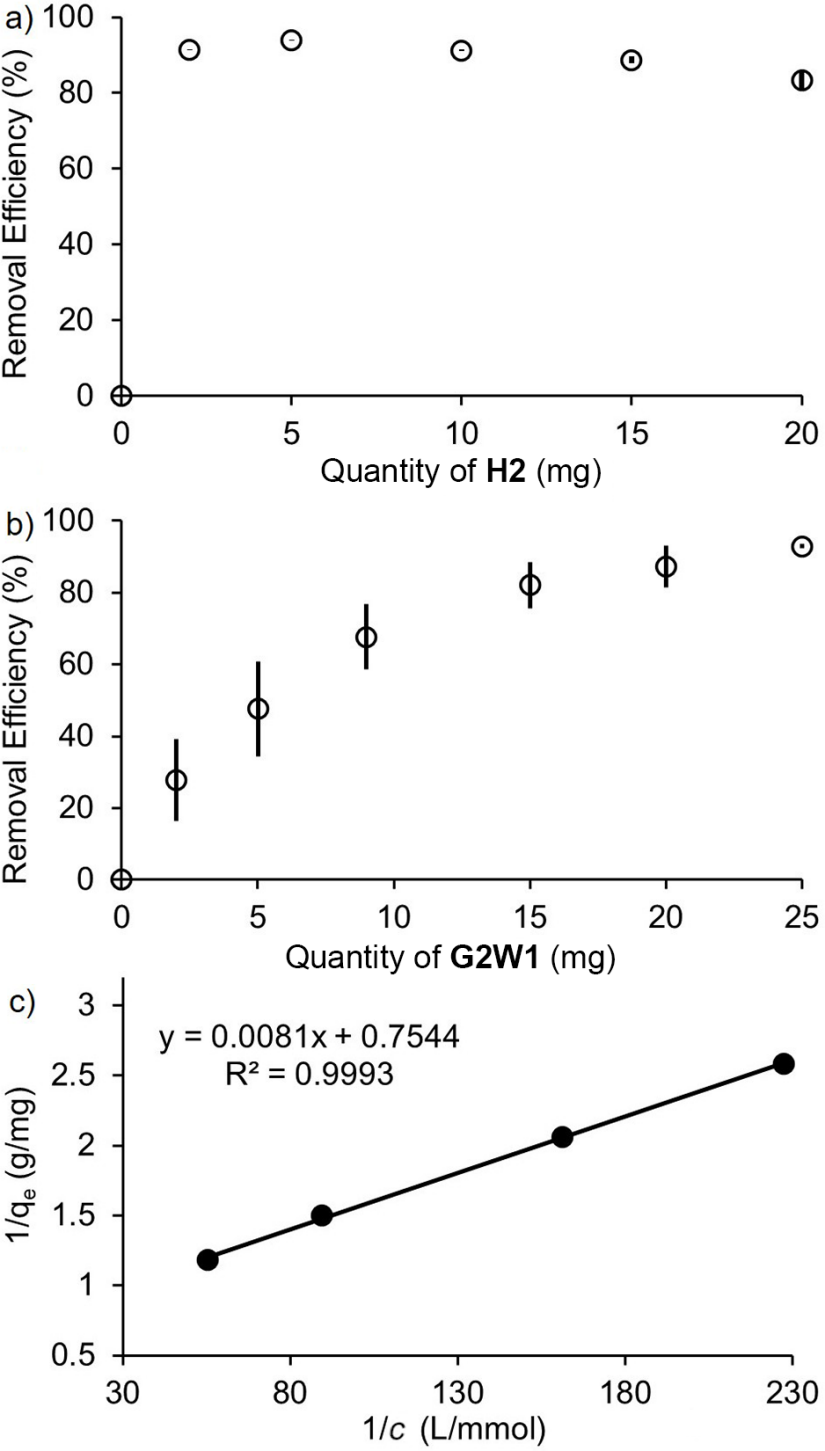
a) Plot of removal efficiency of methylene blue (240 μM, 1 mL) from water after incubating with different amounts of **H2** for 1 hour at 25 °C as determined by UV–vis. b) Plot of removal efficiency of methylene violet (38 μM, 6.4 mL) from water after incubating with different amounts of **G2W1** for 1 h at 25 °C as determined by UV–vis. Experiments were performed in triplicate (*n* = 3). The error bars represent the standard deviation of the three trials. c) The plot of 1/q_e_ versus 1/*c* fitted according to the Langmuir isotherm for **G2W1** with methylene violet.

**Influence of the quantity of host.** Given the very good removal efficiencies of **H2** and **G2W1** for methylene blue and methylene violet, respectively, we first decided to study the influence of the quantity of host **H2** or **G2W1** on the removal efficiency. In a manner analogous to the experiments described above (Figure 4), we incubated different quantities of **H2** and **G2W1** with methylene blue (240 μM, 1 mL) and methylene violet (38 μM, 6.4 mL), respectively, for 1 hour at 25 °C using a ThermoMixer™. The samples were centrifuged, and the supernatants were analyzed by UV–vis spectroscopy. The removal efficiency for each experiment was calculated by using Equation 1. Figure 7a,b shows a plot of removal efficiency versus the quantity of **H2** or **G2W1** used. The **H2**:methylene blue ratio is 30:1 when using **H2** (5.0 mg) and [methylene blue] = 240 μM. The **G2W1**/methylene violet ratio is 30:1 when using **G2W1** (9.0 mg) and 6.4 mL of 38 μM methylene violet. As can be seen in Figure 7a, it is evident that the removal efficiency of **H2** peaks at 94% when 5.0 mg is used and then starts to slowly decrease with higher quantities of **H2**. This is surprising, because, according to the literature, the removal efficiency typically increases with the quantity of the adsorbent until saturation occurs [4,6]. Therefore, the result implies that using only 5.0 mg of **H2** is adequate to saturate all the adsorbent sites of **H2**. In contrast, the removal efficiency of methylene violet gradually increases as the quantity of **G2W1** increases (Figure 7b). To achieve a removal efficiency of 93%, 25.0 mg of **G2W1** must be used. In both cases, removal efficiencies >90% can be achieved by employing an excess of solid host. As described above, we believe that methylene blue is bound within the cavity of **H2**, whereas methylene violet likely stacks on the external face of **G2W1** by π–π interactions. Unfortunately, because the sequestration process occurs from aqueous solution to the solid state, it is not possible to use ^1^H NMR to assess the geometry of the interaction between **H2** or **G2W1** with methylene blue or methylene violet, respectively.

**Determination of the adsorption capacity.** Next, we wanted to learn more about the capacity of **H2** and **G2W1** for the removal of methylene blue and methylene violet. For this, we used adsorption isotherm models. For **H2**, the data from Figure 7a were used to analyze both Langmuir and Freundlich isotherm models. Unfortunately, the data did not fit into either of the two models and gave negative slopes. For **G2W1**, the data from Figure 7b was analyzed and could be successfully fitted into the Langmuir isotherm model. Figure 7c shows a plot of the data fitted using Equation 2, where q_e_ (mg g^−1^) represents the amount of methylene violet adsorbed at equilibrium, *c* (mol L^−1^) denotes the residual methylene violet concentration at equilibrium, and *K* (mol^−1^) indicates the equilibrium constant. According to Figure 7c, the calculated maximum adsorption capacity (q_max,e_) was estimated to be 1.32 mg g^−1^ (R^2^ = 0.9191) which is quite poor and corresponds to a 160:1 ratio of **G2W1**/methylene violet. This indicates that only a small portion of the potential adsorption sites – likely surface sites – are accessible. Since the calculated maximum adsorption capacity and removal efficiencies were significantly lower compared to the adsorbents reported in the literature, we did not conduct any regeneration and reusability experiments for **H2** and **G2W1** [4–6,20].


[2] $$\frac{1}{q_{e}}=\frac{1}{q_{\max,e}}+\frac{1}{q_{\max,e}\cdot K\cdot c}$$

**Figure 8:**
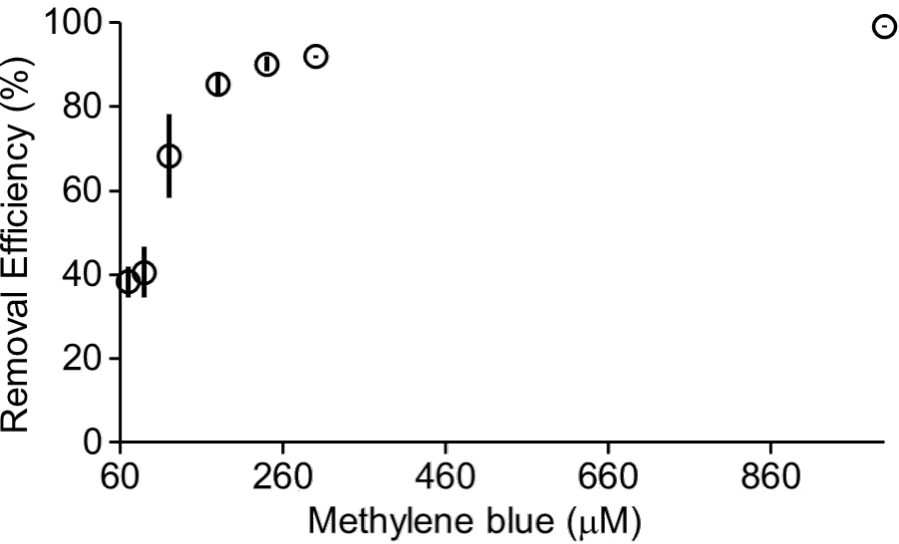
Plot of the removal efficiency versus methylene blue concentration (70, 90, 120, 180, 240, 300, 1000 μM) after incubating with **H2** (5.0 mg) for 1 hour at 25 °C as determined by UV–vis. Experiments were performed in triplicate (*n* = 3). The error bars represent the standard deviation of the three trials.

**Influence of initial methylene blue concentration.** The efficiency of dye removal usually depends on both the initial dye concentration and the number of available sites on the surface of the adsorbent. When the adsorption sites on the adsorbent are saturated, the removal efficiency diminishes as the dye concentration increases. Conversely, when the adsorption sites on the surface of the adsorbent are not saturated, the removal efficiency rises with increasing dye concentration, as higher initial dye concentration creates a stronger mass transfer driving force for adsorption [6]. Therefore, we studied the impact of the initial methylene blue concentration on the removal efficiency of **H2**. For this purpose, we incubated **H2** (5.0 mg, 7.2 μmol) with aqueous solutions of methylene blue (1 mL) at concentrations ranging from 70 μM to 1 mM for 1 hour at 25 °C using a ThermoMixer™. The samples were centrifuged, and the supernatants were analyzed by UV–vis spectroscopy. The removal efficiency values were calculated using Equation 1 and the results are shown in Figure 8. Figure 8 shows that the removal efficiency increases as the initial dye concentration increases. Specifically, we see that at [methylene blue] = 1 mM (**H2**/dye = 7:1), the removal efficiency reaches 99% within 1 hour, while a notable decrease in efficiency (38%) can be seen at an initial methylene blue concentration of 70 μM. However, we did not observe a significant decrease in removal efficiency when the initial methylene blue concentration was between 240 and 1000 μM. This experiment was not conducted with methylene violet due to solubility issues and aggregation of methylene violet at higher concentrations.

**Figure 9:**
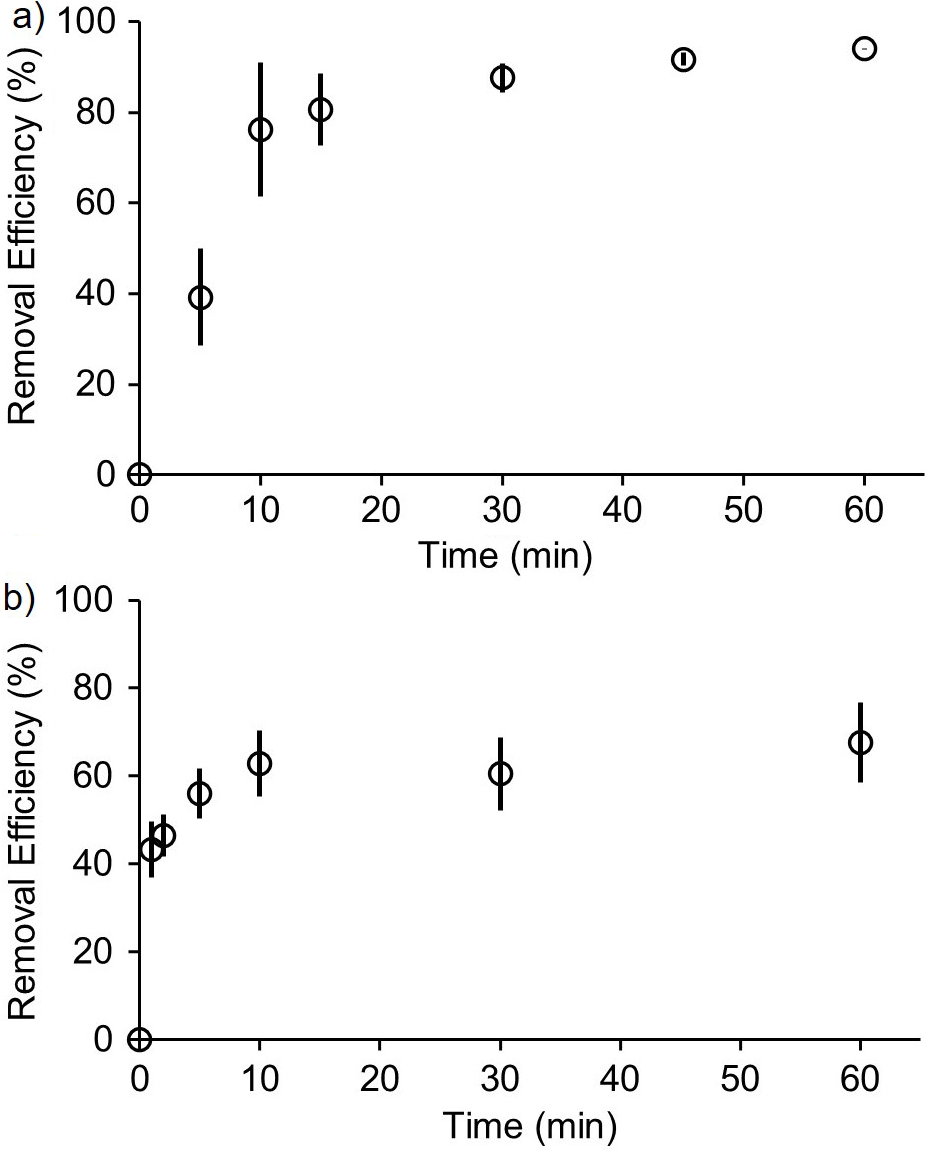
a) Plot of removal efficiency of methylene blue (240 μM, 1 mL) from water as a function of time after incubating with **H2** (5.0 mg) at 25 °C as determined by UV–vis of the supernatant. b) Plot of removal efficiency of methylene violet (38 μM, 6.4 mL) from water as a function of time after incubating with **G2W1** (9.0 mg) at 25 °C as determined by UV–vis of the supernatant. Experiments were performed in triplicate (*n* = 3). The error bars represent the standard deviation of the three trials.

**Time course of removal of methylene blue and methylene violet from water using H2 and G2W1.** Another critical parameter in solid state sequestrants is the amount of time required per cycle. We decided to determine the removal efficiency of **H2** (5.0 mg) and **G2W1** (9.0 mg) at different time intervals for methylene blue (240 μM, 1 mL) and methylene violet (38 μM, 6.4 mL), respectively. For this, we employed a batch-mode experimental design. The results shown in Figure 9 indicate that both methylene blue and methylene violet exhibit rapid uptake and reach saturation within 30 minutes. For methylene blue (Figure 9a), a rapid increase in removal efficiency is observed within the first 10 minutes, likely due to the higher number of available active sites on **H2**. As time progresses and these active sites become saturated, the rate of increase in removal efficiency slows, ultimately reaching a plateau. Similarly, **G2W1** reaches a plateau in removal efficiency within 10 minutes.

## Conclusion

In summary, we have synthesized four new water-insoluble acyclic CB[*n*]-type receptors that possess benzene (**G2W4**), naphthalene (**G2W3**), and triphenylene (**G2W1** and **G2W2**) walls bearing methoxy substituents. The new hosts were fully characterized by ^1^H NMR, ^13^C NMR, IR, mass spectrometry, and X-ray crystallography (**G2W1** and **G2W3**). We studied the efficiency of **G2W1**–**G2W4** along with known comparator host **H2** (OH-substituted) as solid state sequestrants for a panel of five dyes. We found that the new hosts with methoxy substituents (**G2W1**–**G2W4**) are inefficient sequestrants compared to **H2**. The X-ray crystal structures of **G2W1** and **G2W3** show a skewing of their aromatic walls which results in the OMe substituents filling their own cavity which reduces their ability as solid state sequestrants. Solid **H2** is an excellent sequestrant for methylene blue (1 mM) where the removal efficiency reaches 99% when using a 7-fold excess of **H2**. Although we could not quantify the uptake capacity of **H2** for methylene blue, it must be substantially lower than that of previously reported pillar[5]arene (51 mg g^−1^) and calix[4]pyrrole (454 mg g^−1^) polymers [17,20]. Host **G2W1** functions the best as sequestrant for methylene violet achieving an 87% removal efficiency when excess **G2W1** (20 mg) is used. Kinetic studies conducted for both hosts **H2** and **G2W1** in the removal of methylene blue and methylene violet, respectively, demonstrate a rapid sequestration process that reaches a plateau within 30 minutes. In conclusion, this study highlights the importance of avoiding host structural elements that are capable of cavity self-inclusion. Improved function of acyclic CB[*n*] as solid state sequestrants should be possible by their incorporation into porous polymeric materials.

## Experimental

### General experimental details

Starting materials and reagents were purchased from commercial suppliers and were used without further purification. **H2** was synthesized according to a previously published procedure [39]. NMR spectra were measured on commercial spectrophotometers at 400 MHz for ^1^H and 100 MHz for ^13^C in trifluoroacetic acid with a capillary tube containing deuterated water (D_2_O) for locking or in deuterated dimethyl sulfoxide (DMSO-*d*_6_) or in deuterated chloroform (CDCl_3_). Melting points were measured on a Meltemp apparatus in open capillary tubes and are not corrected. IR spectra were recorded on a Thermo Nicolet NEXUS 670 FT/IR spectrometer by attenuated total reflectance (ATR) and are reported in cm^−1^. Mass spectrometry was performed using a JEOL AccuTOF electrospray instrument (ESI). The dye uptake was quantified by UV–vis spectroscopy (Cary 100 Bio UV–visible spectrophotometer) at 25 °C. Incubation of hosts with dyes was performed using an Eppendorf ThermoMixer™ C in 1.5 mL polypropylene tubes or in 15 mL polypropylene tubes. Centrifugation was performed using Eppendorf centrifuges (5804 for synthesis; 5415C for samples from the ThermoMixer™).

### Compound **G2W1**

To a solution of **G2BCE** (362.5 mg, 0.816 mmol) in TFA/Ac_2_O 1:1 (17 mL) was added compound **W1** (1.000 g, 2.447 mmol), and the green solution was heated and refluxed in an oil bath set at 95 °C for 3.5 h under N_2_. The reaction mixture was poured into a flask containing MeOH (280 mL) and stirred overnight at rt. The heterogenous mixture was centrifuged (7500 rpm, 5 min) portionwise, and the supernatant was removed. The crude solid was suspended in MeOH (45 mL) by vortexing and sonicating to dislodge the solid pellet. The solid was collected by centrifugation (7500 rpm, 5 min). This MeOH washing process was repeated twice. The residue was dried under high vacuum to yield a brown solid. The solid was recrystallized by dissolving it in trifluoracetic acid (8 mL), and then MeOH (7 mL) was added dropwise. The solid was collected by centrifugation (7500 rpm, 5 min), and the supernatant was decanted. The wet solid pellet was suspended in MeOH (45 mL) by vortexing and sonicating. The solid was collected by centrifugation (7500 rpm, 5 min), and the supernatant was decanted. This MeOH washing process was repeated twice. The solid was resuspended in MeOH (500 mL) and stirred overnight at rt to remove any remaining TFA. The solid was collected by centrifugation (7500 rpm, 5 min), and the residue was dried under high vacuum to yield **G2W1** as a beige solid (277 mg, 28%). If the brown color remains, repeat the recrystallization. The absence of residual TFA was confirmed by ^19^F NMR. mp >300 °C; ^1^H NMR (400 MHz, DMSO-*d_6_*) δ 8.77 (s, 4H), 7.83 (s, 4H), 5.53 (d, *J* = 15.8 Hz, 2H), 5.43 (d, *J* = 16.1 Hz, 4H), 4.31 (d, *J* = 16.1 Hz, 4H), 4.22 (d, *J* = 15.8 Hz, 2H), 4.03 (s, 12H), 3.72 (s, 12H), 3.37 (s, 12H), 1.78 (s, 6H), 1.72 (s, 6H); ^13^C NMR (100 MHz, CDCl_3_) δ 154.2, 151.4, 148.9, 148.1, 128.5, 124.8, 123.9, 122.2, 108.8, 103.6, 77.4, 75.6, 61.2, 56.1, 55.9, 43.7, 36.1, 17.5, 17.4; IR (ATR, cm^−1^): 2935 (w), 2833 (w), 1714 (w), 1614 (w), 1511 (m), 1456 (m), 1426 (m), 1263 (m), 1238 (s), 1193 (m), 1118 (s), 1073 (m), 1045 (w), 1022 (w), 983 (w); ESIMS (*m/z*): [M + H]^+^ calcd. for C_66_H_69_N_8_O_16_, 1229.48; found, 1229.5.

## Supporting Information

File 1 General experimental details, synthesis and characterization data and spectra of new compounds, procedures for sequestration studies.

## Data Availability

All data that supports the findings of this study are in the published article and/or the supporting information to this article.  The raw data files will be available at the Digital Repository at the University of Maryland (https://drum.lib.umd.edu/home) upon publication at DOI: https://doi.org/10.13016/cyap-ks2r. The x-ray crystal structures of **G2W1 **and **G2W3** are deposited with the Cambridge Crystallographic Data Centre (CCDC 2466610 and CCDC 2466611), respectively.
