# Supplementary material for: Insoluble methylene-bridged glycoluril dimers as sequestrants for dyes
Source: Beilstein J Org Chem. 2025 Oct 29;21:2302–14. doi: 10.3762/bjoc.21.176 (PMC12580995; doi:10.3762/bjoc.21.176)
Supplement: File 1 — General experimental details, synthesis and characterization data and spectra of new compounds, procedures for sequestration studies. [file Beilstein_J_Org_Chem-21-2302-s001.pdf]

BEILSTEIN JOURNAL OF ORGANIC CHEMISTRY

# Supporting Information

for

# Insoluble methylene-bridged glycoluril dimers as sequestrants for dyes

Suvenika Perera, Peter Y. Zavalij and Lyle Isaacs

*Beilstein J. Org. Chem.* **2025,** *21,* 2302–2314. doi:10.3762/bjoc.21.176

# General experimental details, synthesis and characterization data and spectra of new compounds, procedures for sequestration studies

License and Terms: This is a supporting information file under the terms of the Creative Commons Attribution License (https://creativecommons.org/licenses/by/4.0). Please note that the reuse, redistribution and reproduction in particular requires that the author(s) and source are credited and that individual graphics may be subject to special legal provisions.
The license is subject to the *Beilstein Journal of Organic Chemistry* terms and conditions: (https://www.beilstein-journals.org/bjoc/terms)

**Table of contents** Page


General experimental details ........ S2

Data availability statement ........ S2

Synthesis and characterization data for new compounds ........ S3–S7

$^{1}H$ and $^{13}C$ NMR recorded for new compounds ........ S8–S18

Procedure for sequestration of dyes by solid hosts ........ S19–S21

Sequestration of methylene blue and methylene violet using different quantities of **H2** and **G2W1** ........ S21

Sequestration capability of **H2** with different initial methylene blue concentrations ........ S21–S22

Rate of uptake of methylene blue and methylene violet by **H2** and **G2W1** ........ S22

Langmuir isotherm for methylene violet ........ S23

Crystallization of **G2W1** and **G2W3** ........ S24

References ........ S24

**General experimental details.** Starting materials and reagents were purchased from commercial suppliers and were used without further purification. Triphenylenedione was prepared by Echavarren's procedure [1]. 3,3',4,4'-Tetramethoxybiphenyl was prepared according to the literature procedure.[2] **H2** was synthesized according to a previously published procedure.[3] NMR spectra were measured on commercial spectrophotometers at 400 MHz for $^1$H and 100 MHz for $^{13}$C in trifluoroacetic acid with a capillary tube containing deuterated water ($D_2O$) for locking or in deuterated dimethylsulfoxide (DMSO-$d_6$) or in deuterated chloroform ($CDCl_3$). Melting points were measured on a Meltemp apparatus in open capillary tubes and are not corrected. IR spectra were recorded on a Thermo Nicolet NEXUS 670 FT/IR spectrometer by attenuated total reflectance (ATR) and are reported in $cm^{-1}$. Mass spectrometry was performed using a JEOL AccuTOF electrospray instrument (ESI). The dye uptake was quantified by UV–vis spectroscopy (Cary 100 Bio UV–visible spectrophotometer) at 25 °C. Incubation of hosts with dyes was performed using an Eppendorf ThermoMixer™ C in 1.5 mL polypropylene tubes or in 15 mL polypropylene tubes. Centrifugation was performed using Eppendorf centrifuges (5804 for synthesis; 5415C for samples from the ThermoMixer).

**Data availability statement.** All data that supports the findings of this study are in the published article and/or the supporting information to this article. The raw data files will be available at the Digital Repository at the University of Maryland (https://drum.lib.umd.edu/home) upon publication (doi:10.13016/cyap-ks2r). The X-ray crystal structures of **G2W1** and **G2W3** are deposited with the Cambridge Crystallographic Data Centre (CCDC 2466610 and CCDC 2466611), respectively.

**Synthesis and characterization data for new compounds**

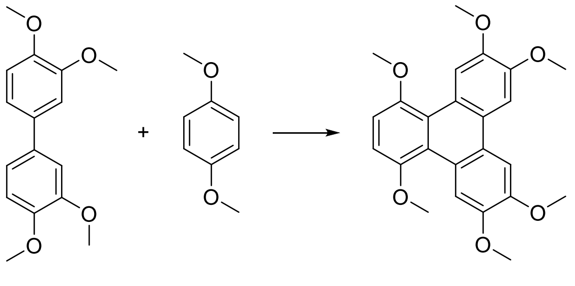
W1

**Compound W1.** To a solution of 3,3',4,4'-tetramethoxy-1,1'-biphenyl [2] (5.00 g, 18.15 mmol) and 1,4-dimethoxybenzene (4.150 g, 30.855 mmol) in dry $CH_2Cl_2$ (108 mL) was added anh. $FeCl_3$ (14.700 g, 90.750 mmol) slowly. The reaction mixture was stirred at rt under $N_2$. The reaction was monitored by TLC (50% hexane in EtOAc, $R_f$ 0.3). After 4 days, the reaction mixture was diluted with $CH_2Cl_2$ (350 mL) and washed with 2 M KOH (500 mL). The aqueous layer was back extracted with $CH_2Cl_2$ (300 mL). The combined organic layer was filtered through a Büchner funnel and washed with excess $CH_2Cl_2$. The filtrate was washed with brine (200 mL) and water (200 mL), respectively. If the organic layer still contains solid particles, repeat the vacuum filtration. The organic layer was concentrated after drying with anh. $Na_2SO_4$. The crude solid was purified by column chromatography ($SiO_2$, 50% EtOAc in hexane) to yield **W1**, a light pink solid (2.562 g, 35%). M.p. 184-187 ˚C. $^{1}$H NMR (400 MHz, $CDCl_3$): δ 9.21 (s, 2H), 7.80 (s, 2H), 7.12 (s, 2H), 4.13 (s, 6H), 4.07 (s, 6H), 4.02 (s, 6H). $^{13}$C NMR (100 MHz, $CDCl_3$): δ 152.5, 148.7, 147.6, 125.3, 123.2, 110.9, 110.4, 104.3, 103.7, 57.2, 56.0, 55.8. IR (ATR, $cm^{-1}$): 2935w, 2833w, 1714w, 1614w, 1511m, 1456m, 1426m, 1263m, 1238s, 1193m, 1073m, 1045w, 1022m, 983m. MS (ESI): *m/z* calcd. for (C24H25O6, [M+H]+), 409.17; found 409.2 ([M+H] $^{+}$).

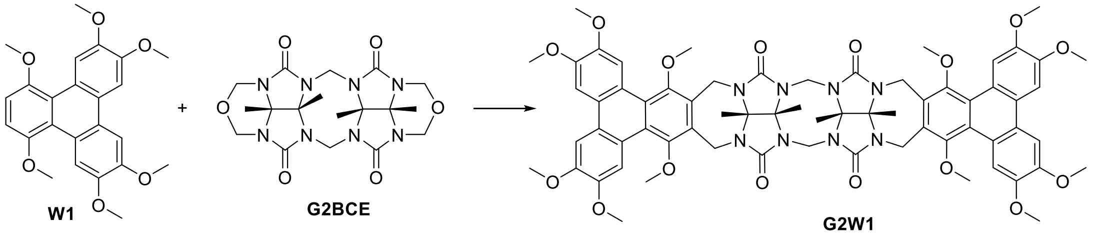


**Host G2W1.** To a solution of **G2BCE** (362.5 mg, 0.816 mmol) in TFA/$Ac_2O$ 1:1 (17 mL) was added compound **W1** (1 g, 2.447 mmol), and the green solution was heated and refluxed in an oil bath set at 95 °C for 3.5 h under $N_2$. The reaction mixture was poured into a flask containing MeOH (280 mL) and stirred overnight at rt. The mixture was centrifuged (7500 rpm, 5 min), and the supernatant was removed. The solid was suspended in MeOH (45 mL) by vortexing and

sonicating to dislodge the solid pellet. The solid was collected by centrifugation (7500 rpm, 5 min), and this MeOH rinsing process was repeated two times in total. The residue was dried under a high vacuum to yield a brown solid. The solid was recrystallized by dissolving it in trifluoracetic acid (8 mL), and then MeOH (7 mL) was added dropwise. The solid was collected by centrifugation (7500 rpm, 5 min), and the supernatant was decanted. The wet solid pellet was suspended in MeOH (45 mL) by vortexing and sonicating. The solid was collected by centrifugation (7500 rpm, 5 min), and the supernatant was decanted. This washing process was repeated twice. The solid was resuspended in MeOH (500 mL) and stirred overnight at rt to remove any remaining TFA. The solid was collected by centrifugation (7500 rpm, 5 min), and the residue was dried under high vacuum to yield **G2W1** as a beige-colored solid (277 mg, 28%). If the brown color remains, repeat the recrystallization. The removal of TFA was confirmed by $^{19}$F NMR. M.p. > 300 ˚C. $^{1}$H NMR (400 MHz, DMSO-$d_6$): δ 8.77 (s, 4H), 7.83 (s, 4H), 5.53 (d, *J* = 15.8 Hz, 2H), 5.43 (d, *J* = 16.1 Hz, 4H), 4.31 (d, *J* = 16.1 Hz, 4H), 4.22 (d, *J* = 15.8 Hz, 2H), 4.03 (s, 12H), 3.72 (s, 12H), 3.37 (s, 12H), 1.78 (s, 6H), 1.72 (s, 6H). $^{13}$C NMR (100 MHz, $CDCl_3$): δ 154.2, 151.4, 148.9, 148.1, 128.5, 124.8, 123.9, 122.2, 108.8, 103.6, 77.4, 75.6, 61.2, 56.1, 55.9, 43.7, 36.1, 17.5, 17.4. IR (ATR, cm$^{-1}$): 2935w, 2833w, 1714w, 1614w, 1511m, 1456m, 1426m, 1263m, 1238s, 1193m, 1118s, 1073m, 1045w, 1022w, 983w. MS (ESI): *m/z* calcd. for (C66H69N8O16, [M+H]+), 1229.48; found 1229.5 ([M+H]$^{+}$).

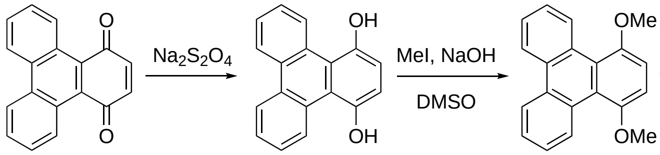

W2

**Compound W2.** To a solution of triphenylene-1,4-dione (199 mg, 0.7705 mmol) in ether (16 mL) was added 10% aqueous $Na_2S_2O_4$ (78 mL, 44.691 mmol). The mixture was stirred at 30 °C for 6.5 h under $N_2$. The mixture was washed with ethyl acetate (25 mL × 3) and the organic layer was washed with brine (25 mL). The organic layer was concentrated after drying with anh. $Na_2SO_4$ to obtain triphenylene-1,4-diol as an orange solid (184 mg, 92%) that is highly prone to oxidation. $^{1}$H NMR (400 MHz, DMSO-$d_6$): δ 9.85 (s, 2H), 9.72 (d, J = 7.9 Hz, 2H), 8.65 (d, J = 8.5 Hz, 2H), 7.58-7.54 (m, 4H), 7.06 (s, 2H). To a solution of triphenylene-1,4-diol (956 mg, 3.667 mmol) in DMSO (11.4 mL) was added NaOH (440 mg, 11.000 mmol) and iodomethane (0.68 mL, 11.000 mmol). The resulting mixture was stirred at rt for two days, open to air. The reaction mixture was poured

into an ice/water mixture (≈115 mL) and stirred at rt for 2 h. The precipitate was obtained by centrifugation (7500 rpm, 5 min), and the supernatant was decanted. The crude solid was purified by column chromatography ($SiO_2$, 98:2 petroleum ether/ethyl acetate) to give **W2** as a light pink solid (313 mg, 31%). The $^1$H NMR spectrum matches that reported in the literature [3].

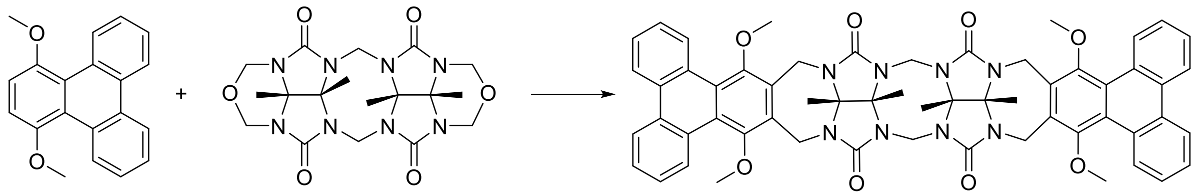


**Host G2W2**. To a solution of **G2BCE** (100 mg, 0.223 mmol) in TFA/$Ac_2O$ 1:1 (5 mL) was added 1,4-dimethoxytriphenylene (**W2**, 192.8 mg, 0.669 mmol), and the solution was heated and refluxed in an oil bath set at 95 °C for 3.5 h under $N_2$. The reaction mixture was poured into MeOH (100 mL) and stirred at rt overnight. The mixture was centrifuged (7500 rpm, 5 min), and the supernatant was decanted and concentrated. Acetone (5 mL) was added to the solid residue and stirred at rt for 30 minutes. The white precipitate was obtained by centrifugation (7500 rpm, 5 min). The residue was dried under high vacuum to yield **G2W2** as a white solid (73 mg, 33%). M.p. > 300 ˚C. $^1$H NMR (400 MHz, DMSO-$d_6$): δ 9.12 (d, $J$ = 7.8 Hz, 4H), 8.55 (d, $J$ = 7.8 Hz, 4H), 7.61 (t, $J$ = 7.8 Hz, 4H), 7.46 (t, $J$ = 7.8 Hz, 4H), 5.50 (d, $J$ = 15.8 Hz, 2H), 5.38 (d, $J$ = 16.0 Hz, 4H), 4.32 (d, $J$ = 16.0 Hz, 4H), 4.20 (d, $J$ = 15.8 Hz, 2H), 3.25 (s, 12H), 1.76 (s, 6H), 1.71 (s, 6H). $^{13}$C NMR (100 MHz, $CDCl_3$): δ 154.3, 151.9, 130.1, 129.7, 128.5, 127.4, 127.2, 127.1, 124.8, 123.0, 77.3, 75.6, 61.2, 43.7, 36.0, 17.4, 17.3. IR (ATR, cm$^{-1}$): 2972w, 2938w, 2830w,1726s, 1453m, 1427m, 1379m, 1341m, 1303s, 1255m, 1201w, 1086m, 1072m, 1041m, 1000m, 954m, 924m. MS (ESI): *m/z* calcd. for (C58H53N8O8, [M+H]+), 989.40; found, 989.6 ([M+H]$^+$).

**Compound W3.** To a solution of naphthalene-1,4-diol (916 mg, 5.718 mmol) in DMSO (18 mL) was added NaOH (687 mg, 17.156 mmol) and iodomethane (1.1 mL, 17.156 mmol). The resulting mixture was stirred at rt for two days, open to air. The reaction mixture was poured into an ice/water mixture (≈180 mL) and stirred at rt for 2 h. The precipitate was obtained by centrifugation (7500 rpm, 5 min), and the supernatant was decanted. The solid was dried under

high vacuum to give **W3** as a light pink solid (935 mg, 87%). The $^{1}$H NMR data matches that reported in the literature [4].

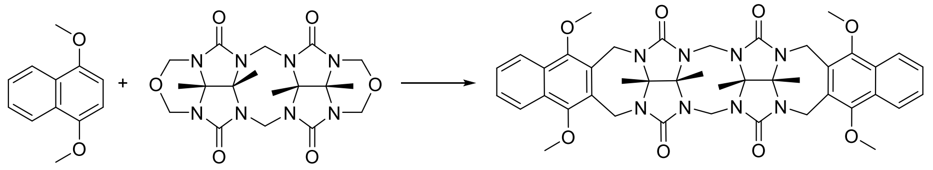

W3 G2BCE G2W3

**Host G2W3.** To a solution of **G2BCE** (930 mg, 2.072 mmol) in TFA/$Ac_2O$ 1:1 (15 mL) was added 1,4-dimethoxynaphthalene (**W3**, 739 mg, 6.216 mmol). The mixture was heated and refluxed in an oil bath set at 70 °C under $N_2$ for 3.5 h. The reaction mixture was poured into MeOH (350 mL), and then water (10 mL) was added. The mixture was stirred overnight at rt. The precipitate was collected by centrifugation (8000 rpm, 10 min). The solid was suspended in MeOH (80 mL) and sonicated, and vortexed to dislodge the pellet. The precipitate was collected by centrifugation (8000 rpm, 10 min). This process was repeated until the supernatant became colorless. The solid was dried under high vacuum to yield **G2W3** as a light pink solid (967 mg, 59%). M.p. > 300 ˚C. $^{1}$H NMR (400 MHz, DMSO-$d_6$): δ 8.00-7.40 (m, 8H), 5.43 (d, $J$ = 15.7 Hz, 2H), 5.20 (d, $J$ = 15.9 Hz, 4H), 4.25 (d, $J$ = 15.9 Hz, 4H), 4.17 (d, $J$ = 15.7 Hz, 2H), 3.53 (s, 12H), 1.72 (s, 6H), 1.67 (s, 6H). $^{13}$C NMR δ (100 MHz, DMSO-$d_6$): 153.8, 149.1, 127.5, 127.2, 126.4, 122.7, 77.1, 75.4, 62.1, 43.5, 35.6, 16.9, 16.5. IR (ATR, cm$^{-1}$): 2977w, 2950w, 2844w, 1733s, 1454s, 1431s, 1354s, 1304s, 1238m, 1197m, 1095m, 1079m, 1042m, 997m, 962m, 952m. MS (ESI): *m/z* calcd. for (C42H45N8O8, [M+H]+), 789.34; found 789.5 ([M+H]$^{+}$).

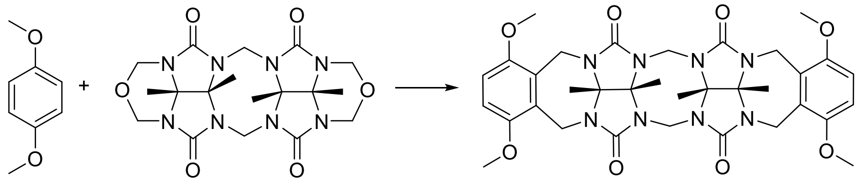

G2BCE G2W4

**Host G2W4**. To a solution of **G2BCE** (216.4 mg, 0.482 mmol) in TFA/$Ac_2O$ 1:1 (3.6 mL) was added 1,4-dimethoxybenzene (200 mg, 1.447 mmol). The mixture was heated and refluxed in an oil bath set at 70 °C under $N_2$ for 3.5 h. The reaction mixture was poured into MeOH (50 mL) and stirred for 1 h at rt. The precipitate was collected by centrifugation (8000 rpm, 5 min). The solid was suspended in MeOH (40 mL) and sonicated, and vortexed to dislodge the pellet. The precipitate was collected by centrifugation (8000 rpm, 5 min). The solid was dried under high vacuum to give **G2W4** as a white solid

(205 mg, 62%). M.p. > 300 ˚C. $^{1}H$ NMR (400 MHz, DMSO-$d_6$): δ 6.81 (s, 4H), 5.47 (d, *J* = 15.7 Hz, 2H), 5.27 (d, *J* = 16.2 Hz, 4H), 4.12 (d, *J* = 15.7 Hz, 4H), 4.00 (d, *J* = 16.2 Hz, 4H), 3.70 (s, 12H), 1.71 (s, 6H), 1.63 (s, 6H). $^{13}C$ NMR (100 MHz, TFA with $D_2O$ cap.): δ 160.1, 153.2, 127.8, 117.1, 83.5, 80.9, 59.5, 46.4, 38.0, 17.3, 17.2. IR (ATR, $cm^{-1}$): 2987w, 2943w, 2836w, 1721s, 1456s, 1298m, 1257m, 1094m, 1075m, 1003w, 950w. MS (ESI): *m/z* calcd. for (C34H41N8O8, [M+H]+), 689.31; found 689.4 ([M+H] $^{+}$).

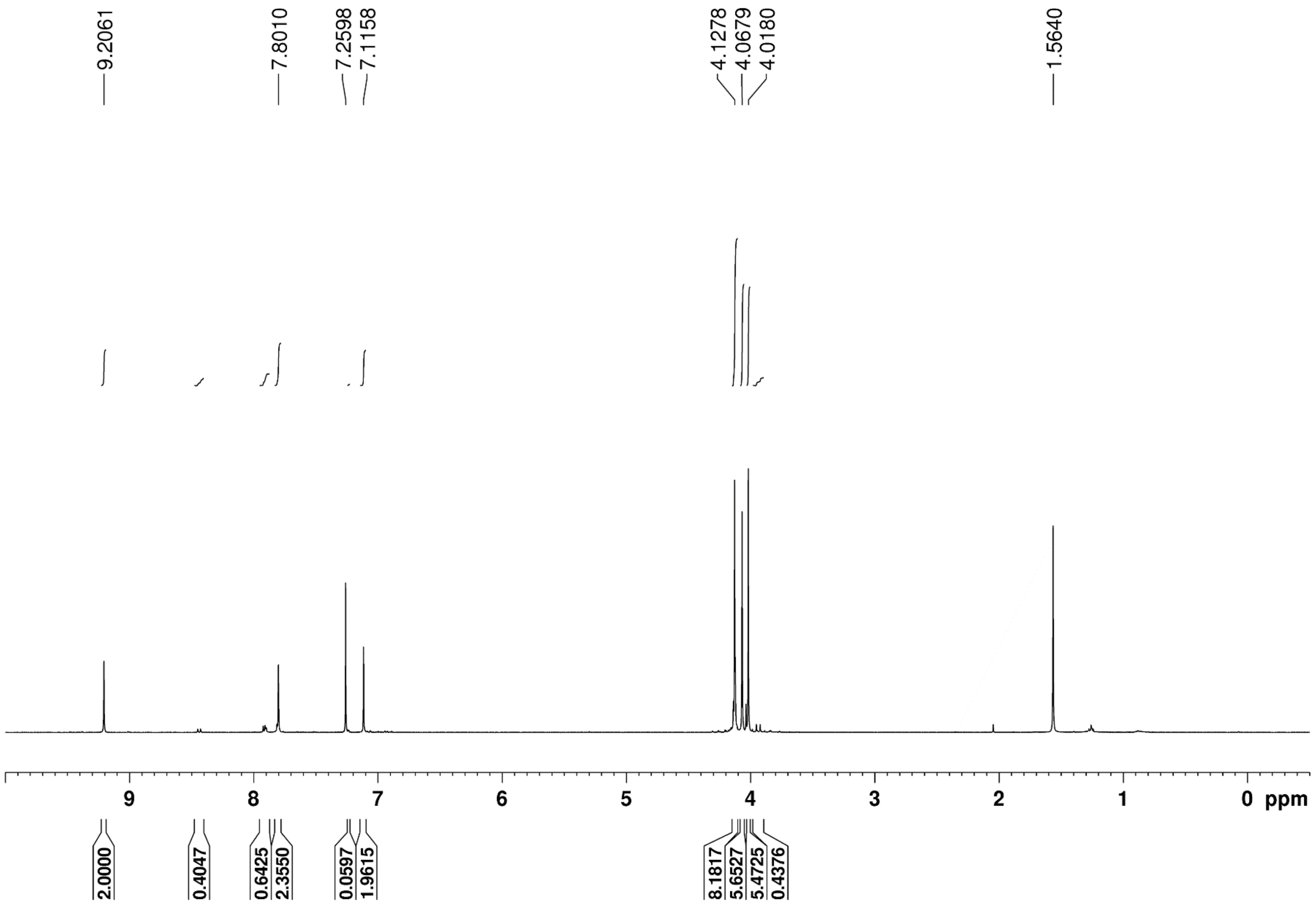


**Figure S1.** $^{1}$H NMR spectrum recorded (400 MHz, $CDCl_3$) for **W1**.

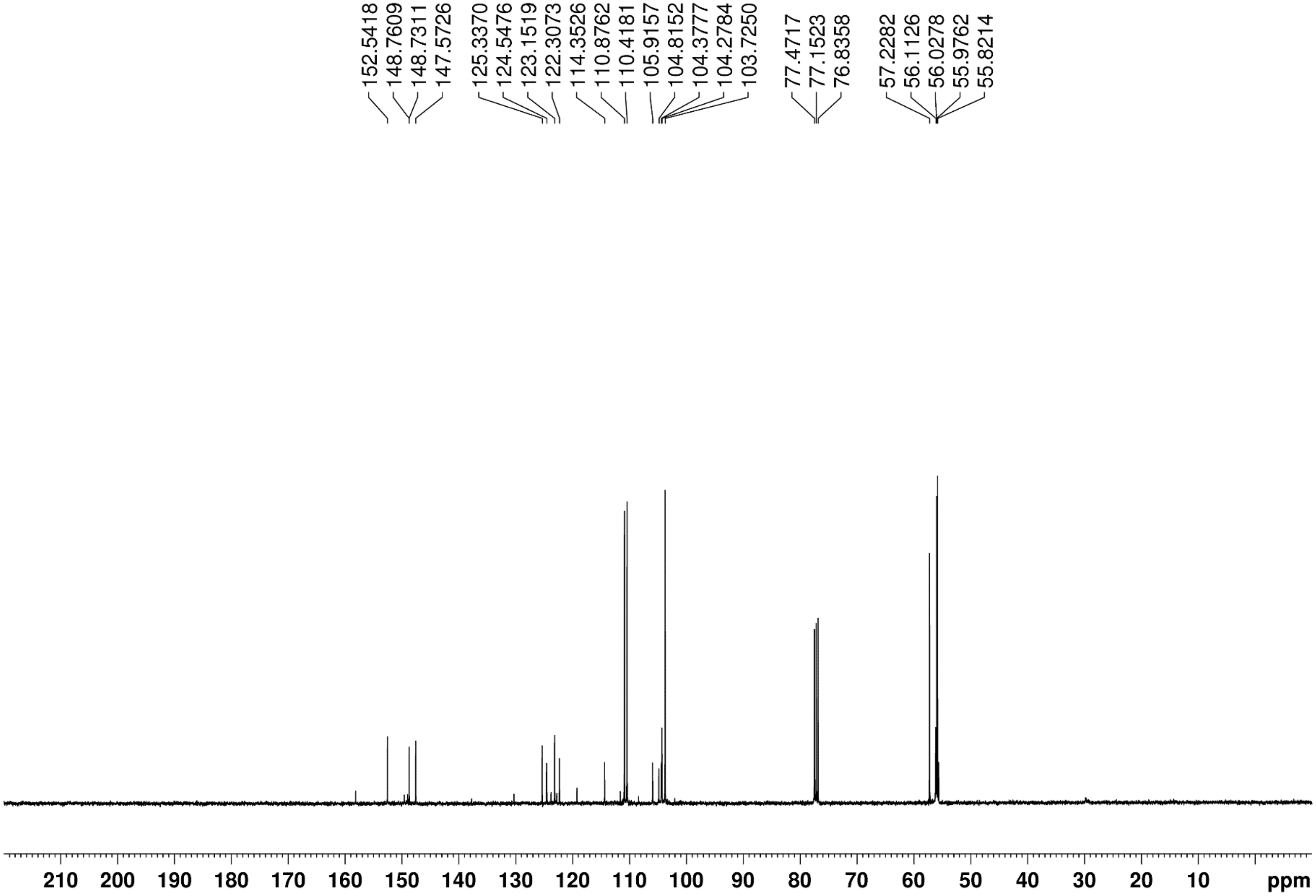


**Figure S2.** $^{13}C$ NMR spectrum recorded (100 MHz, $CDCl_3$) for **W1**.

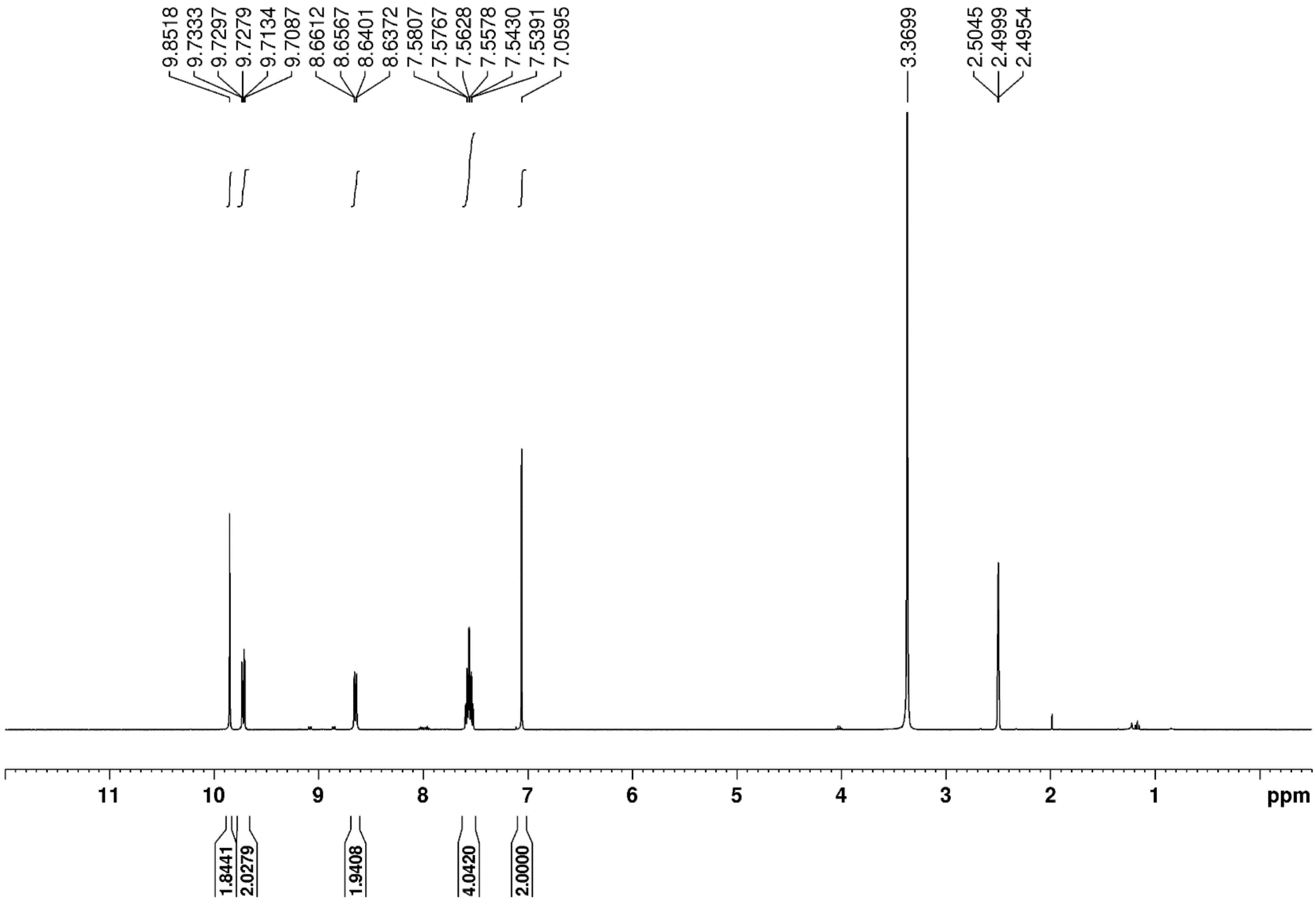


**Figure S3.** $^{1}H$ NMR spectrum recorded (400 MHz, DMSO-$d_6$) for triphenylene-1,4-diol.

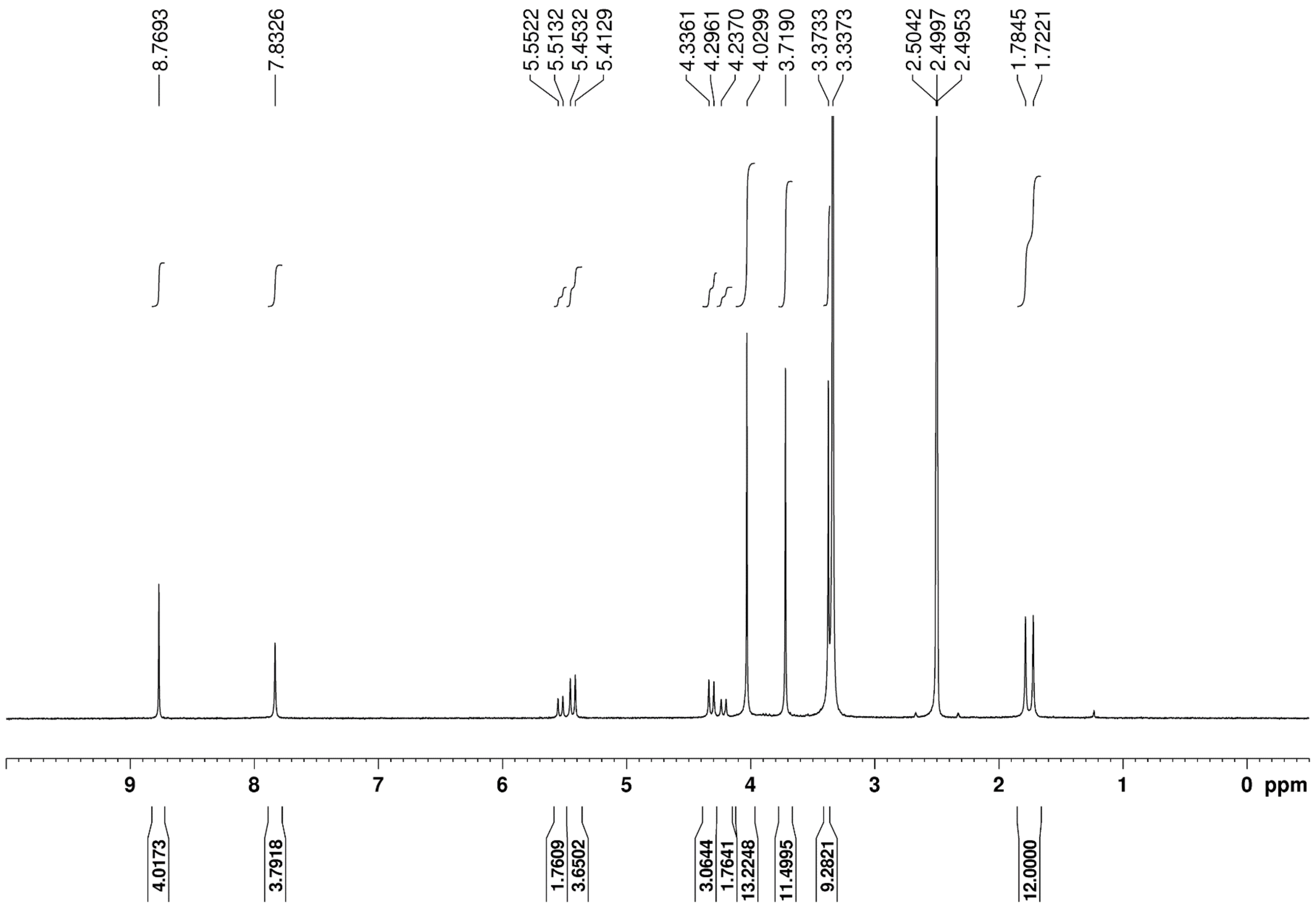


**Figure S4.** $^{1}$H NMR spectrum recorded (400 MHz, DMSO-$d_6$) for **G2W1**.

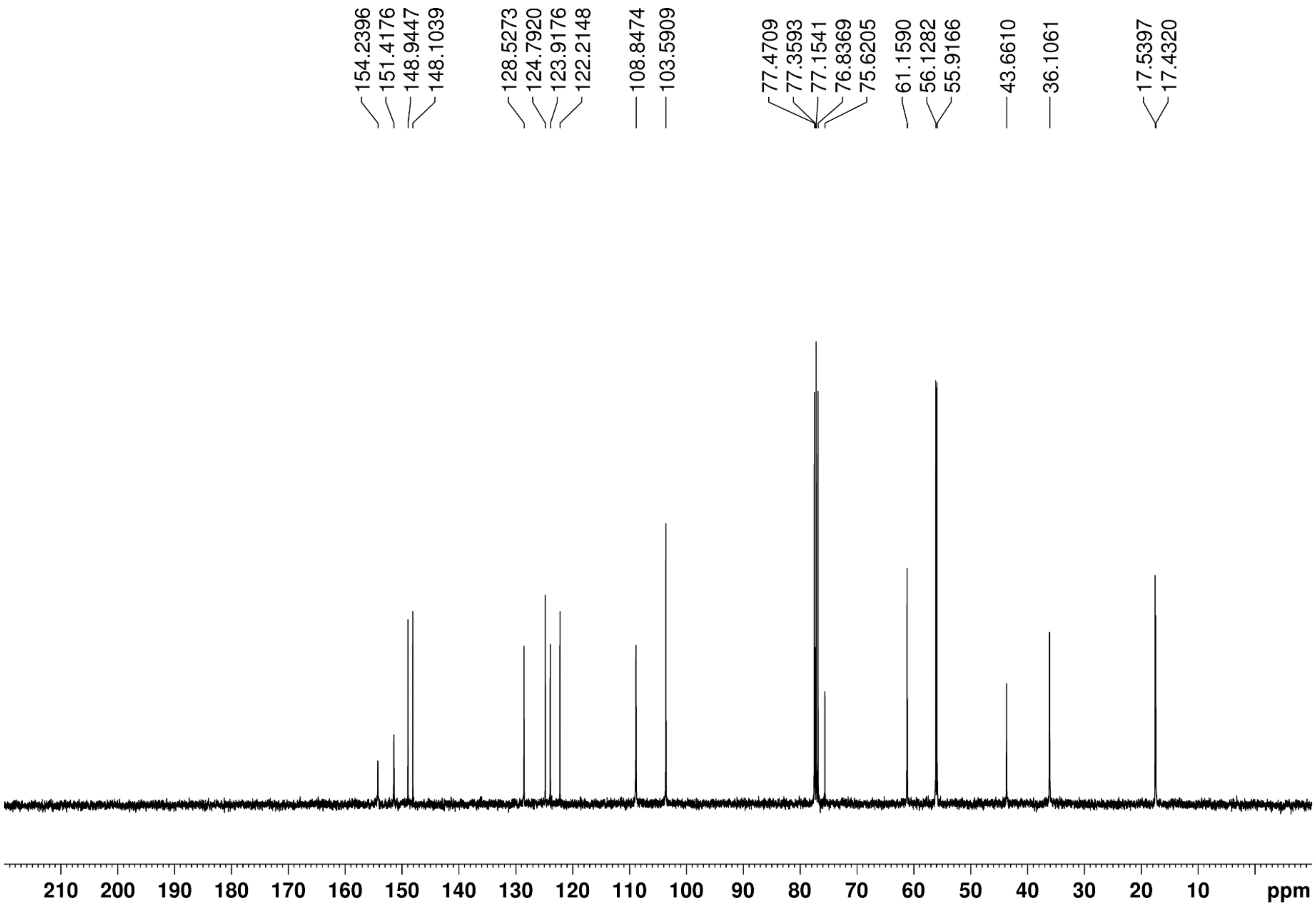


**Figure S5.** $^{13}C$ NMR spectrum recorded (100 MHz, $CDCl_3$) for **G2W1**.

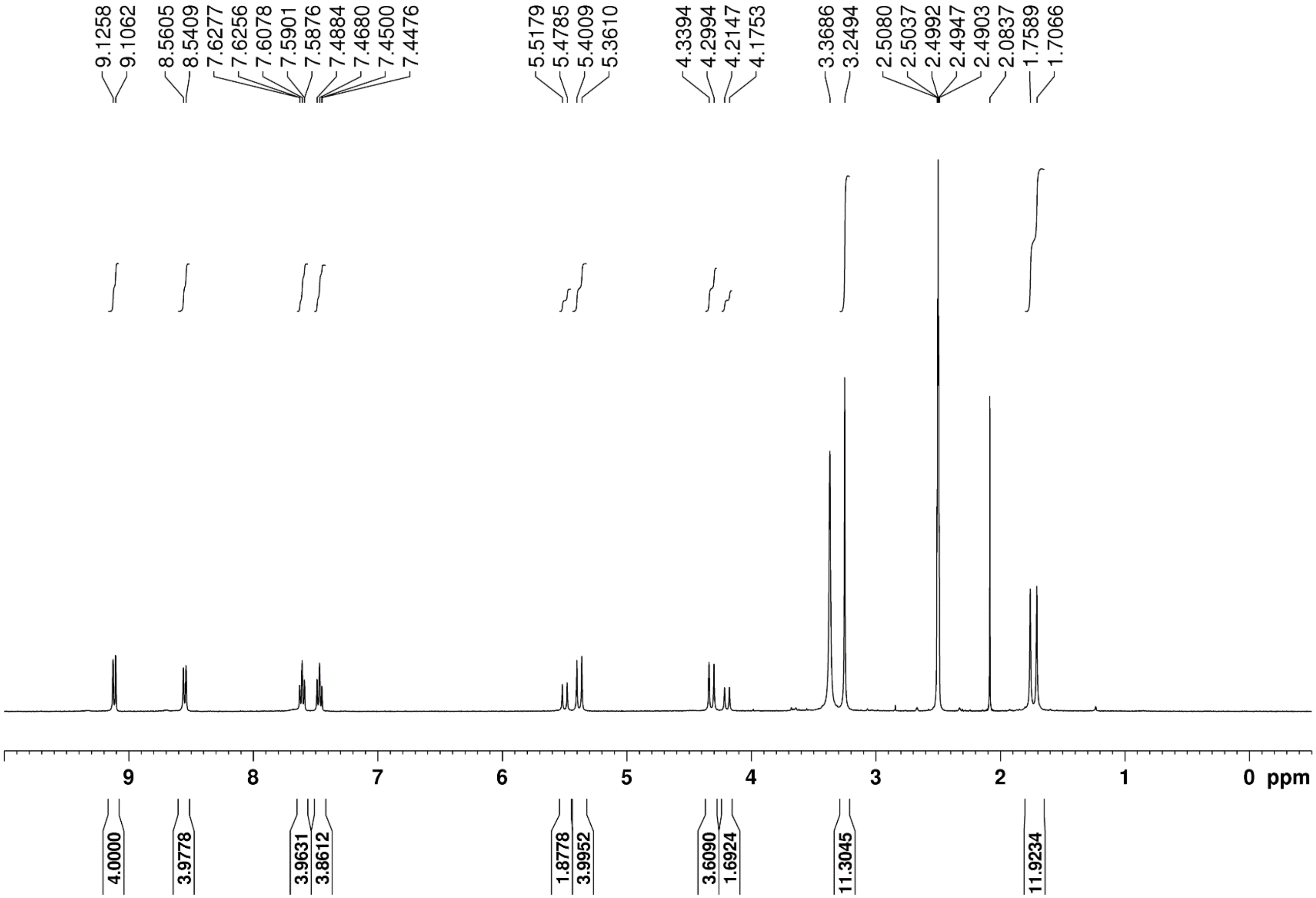


**Figure S6.** $^{1}$H NMR spectrum recorded (400 MHz, DMSO-$d_6$) for **G2W2**.

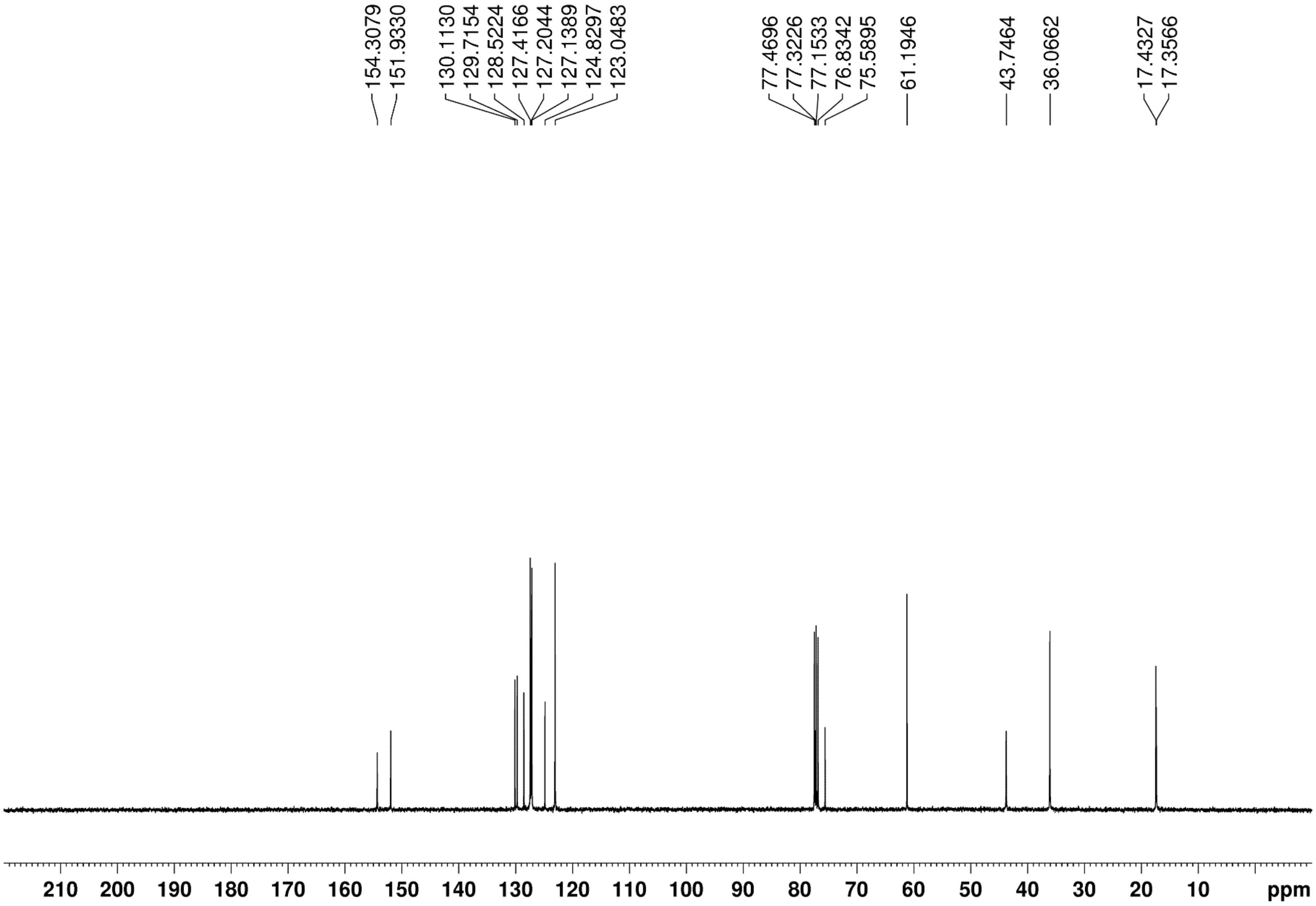


**Figure S7.** $^{13}C$ NMR spectrum recorded (100 MHz, $CDCl_3$) for **G2W2**.

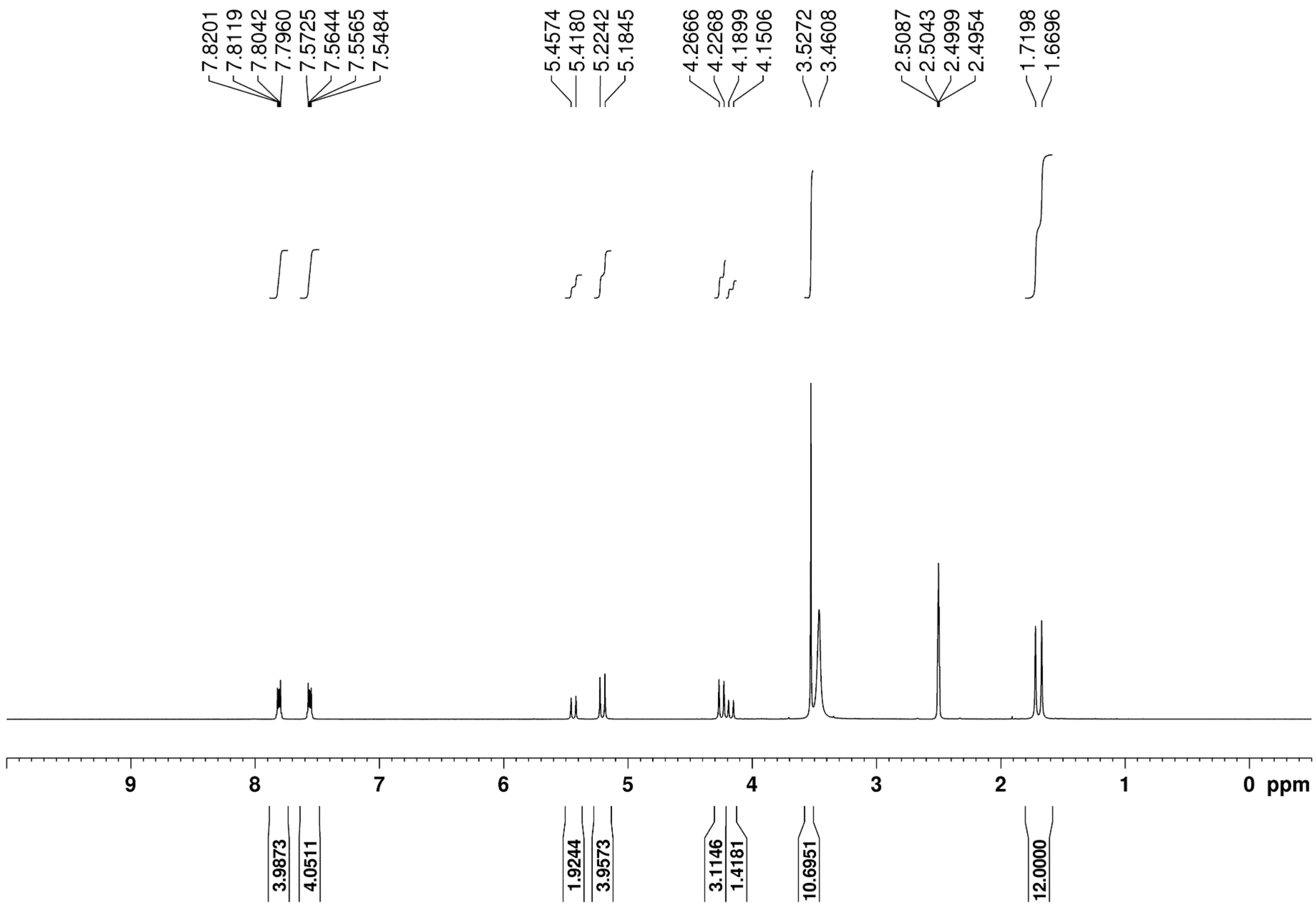


**Figure S8.** $^{1}$H NMR spectrum recorded (400 MHz, DMSO-$d_6$) for **G2W3**.

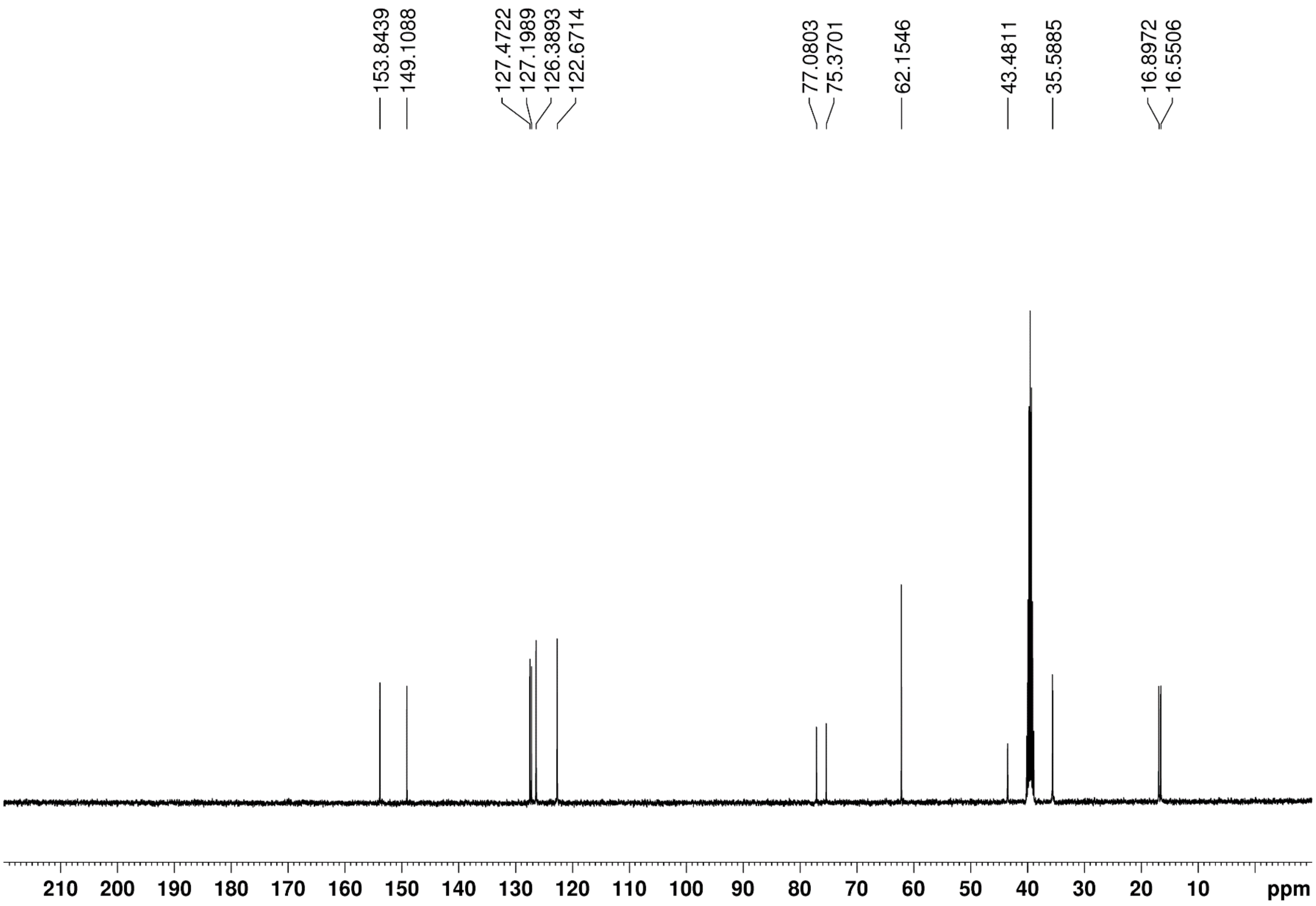


**Figure S9.** $^{13}C$ NMR spectrum recorded (100 MHz, DMSO-$d_6$) for **G2W3**.

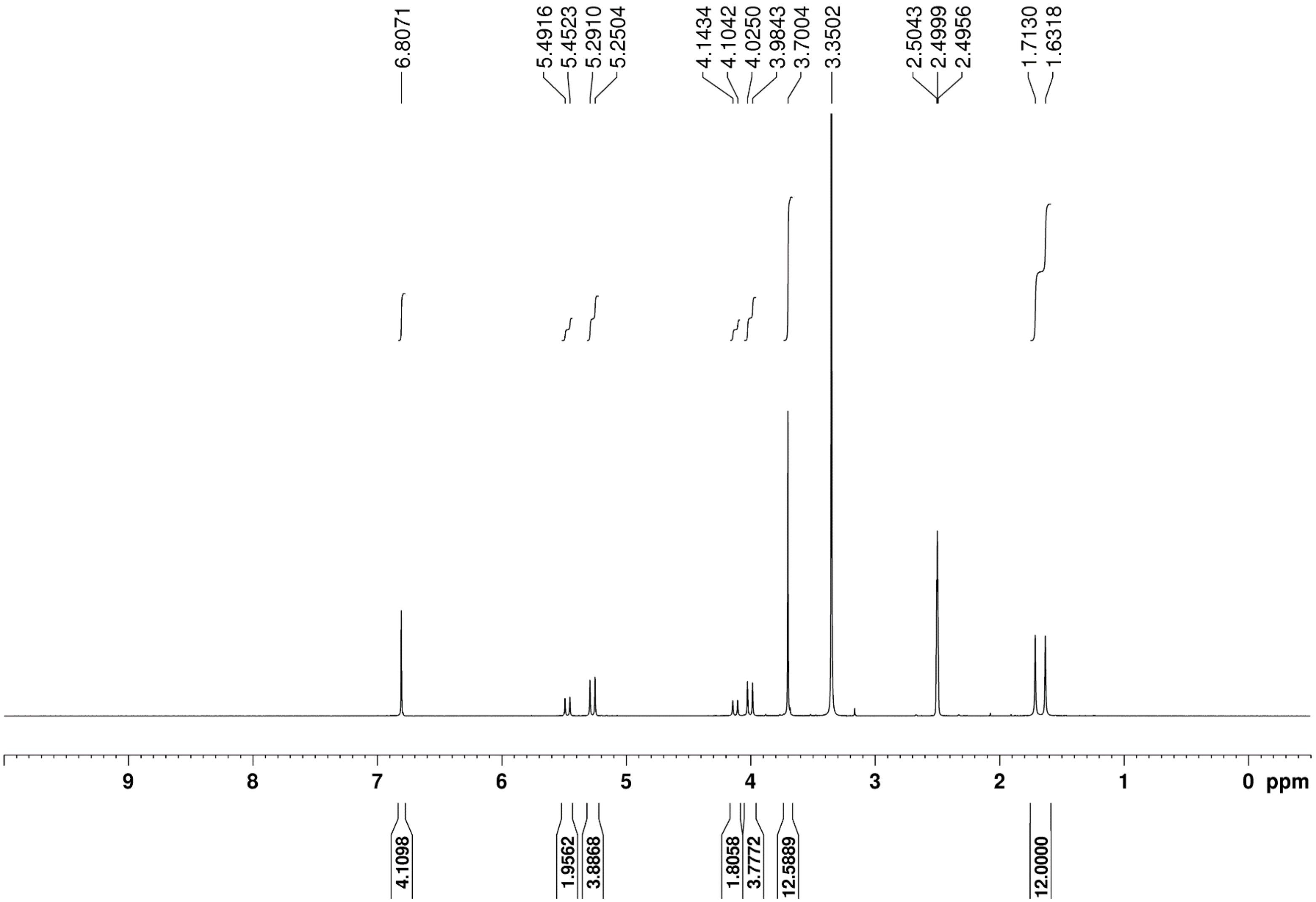


**Figure S10.** $^1$H NMR spectrum recorded (400 MHz, DMSO-$d_6$) for **G2W4**.

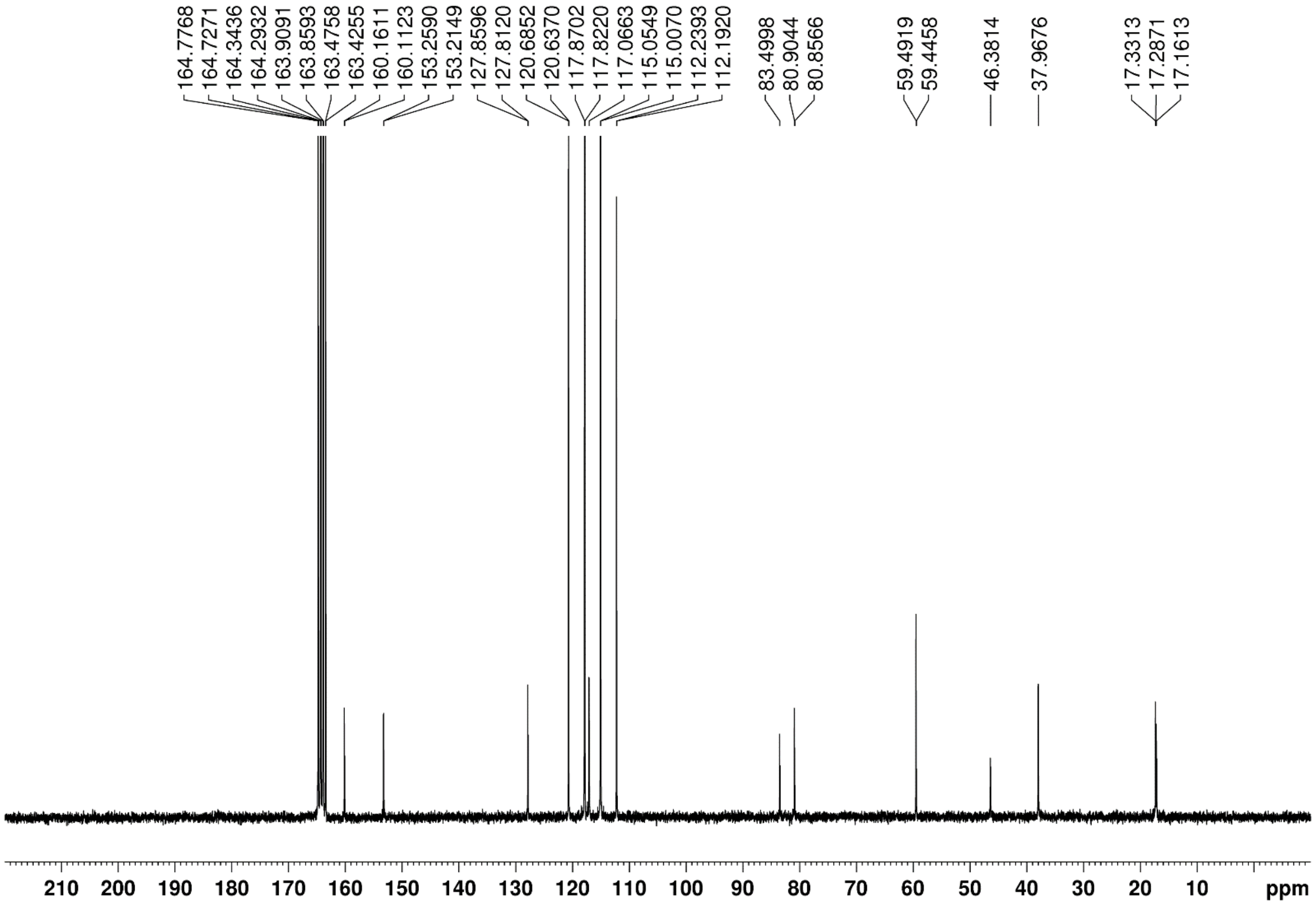


**Figure S11.** $^{13}$C NMR spectrum recorded (100 MHz, TFA, $D_2O$ capillary) for **G2W4**.

**Procedure for the sequestration of methylene blue, rhodamine 6G, methyl violet 6B, acridine orange, and methylene violet by H2, G2W1, G2W2, G2W3, and G2W4.** The five hosts (7.2 µmol each; **H2** (5.0 mg), **G2W1** (9.0 mg), **G2W2** (7.2 mg), **G2W3** (5.7 mg), and **G2W5** (5.0 mg)) were incubated separately with aq. solutions of methylene blue (240 µM, 1 mL) or rhodamine 6G (240 µM, 1 mL) or methyl violet 6B (240 µM, 1 mL) or acridine orange (100 µM, 2.4 mL) or methylene violet (38 µM, 6.4 mL) at 25 °C for 1 h using the ThermoMixer™. Then, the mixtures were centrifuged (11,000 rpm, 10 min) (for acridine orange and methylene violet; 7500 rpm, 5 min), and the supernatants were analyzed by UV–vis spectroscopy. The removal efficiency was determined using Equation S1 where $c_0$ and $c_t$ are the dye concentrations before and after adsorptive sequestration. Figure S11 shows the removal efficiency achieved by each host for each dye. Figure S12 shows the calibration curves used to determine the dye concentration in each experimental sample.

$$\text{Removal efficiency (\%)} = \frac{C_0 - C_t}{C_0} \times 100 \qquad \text{(S1)}$$

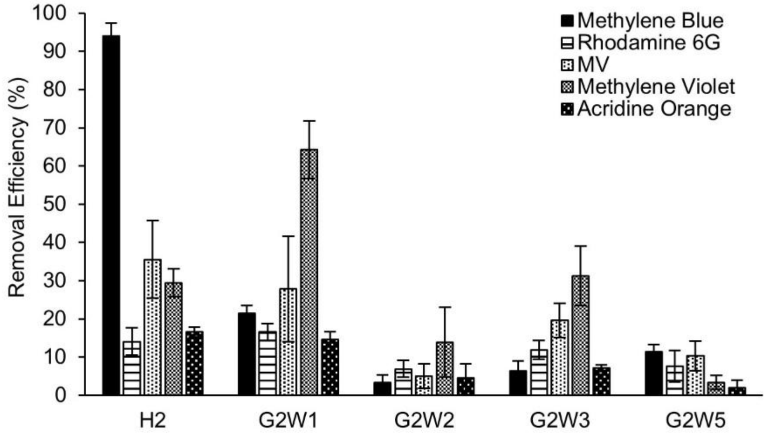


**Figure S12**. Plot of removal efficiency achieved by each host for each dye.

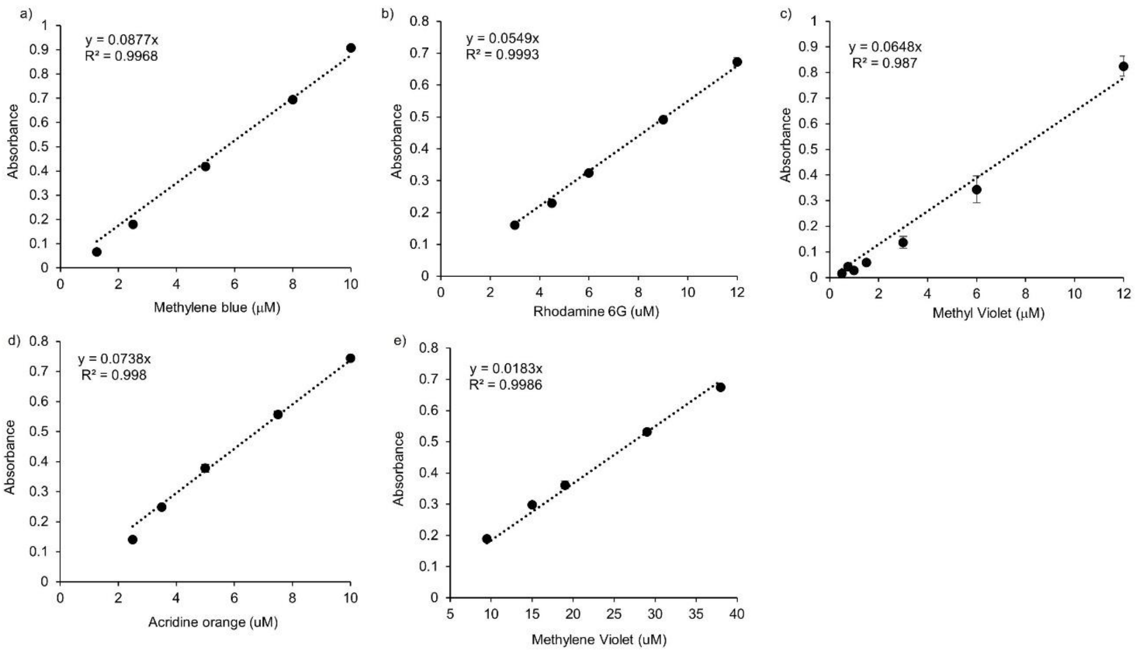


**Figure S13.** Calibration curves constructed using a series of samples of known [dye]: a) methylene blue, b) rhodamine 6G, c) methyl violet 6B, d) acridine orange, e) methylene violet.

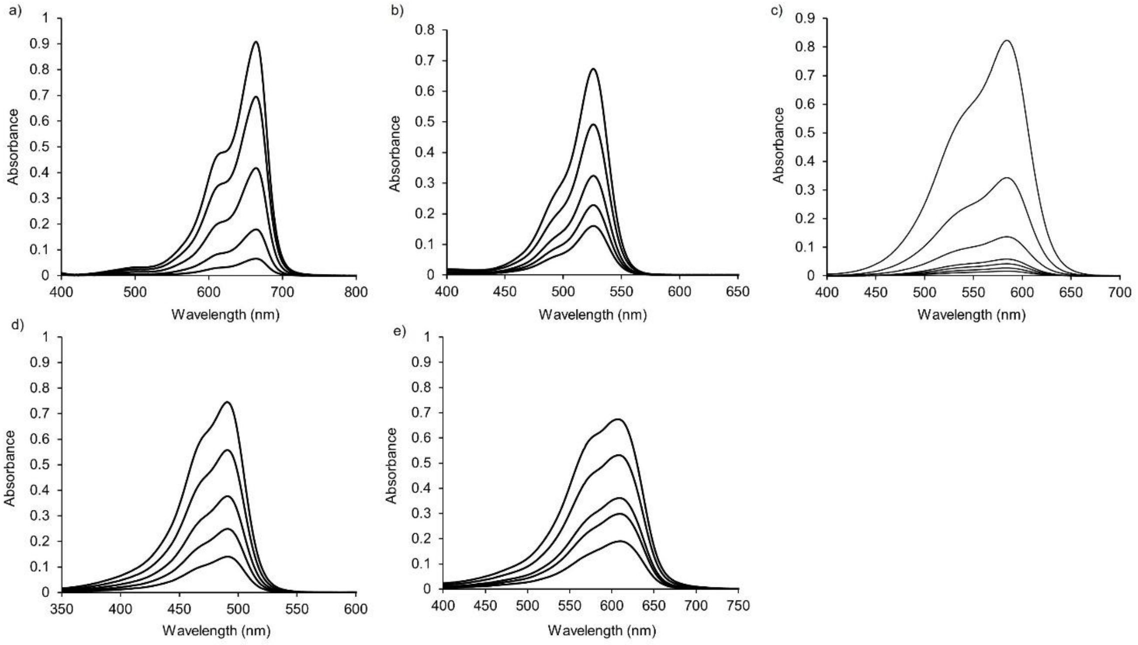


**Figure S14.** UV–vis traces for known [dye] used to construct the calibration curves; a) methylene blue (10, 8, 5, 2.5, 1.25 μM), b) rhodamine 6G (12, 9, 6, 4.5, 3 μM), c) methyl violet 6B (12, 6, 3, 1.5, 1, 0.75, 0.5 μM), d) acridine orange (10, 7.5, 5, 3.5, 2.5 μM), e) methylene violet (38, 29, 19, 15, 9.5 μM).

**Sequestration of methylene blue and methylene violet using different quantities of H2 and G2W1, respectively.** Methylene blue (240 μM, 1 mL) was incubated with different amounts of **H2** for 1 h at rt using the ThermoMixer™. The mixtures were centrifuged (11,000 rpm, 10 min), and the supernatants were analyzed by UV–vis spectroscopy. Methylene violet (38 μM, 6.4 mL) was incubated with different amounts of **G2W1** for 1 h at rt using the ThermoMixer™. The mixtures were centrifuged (7500 rpm, 5 min), and the supernatants were analyzed by UV–vis spectroscopy. The removal efficiency was determined by using Equation S1.

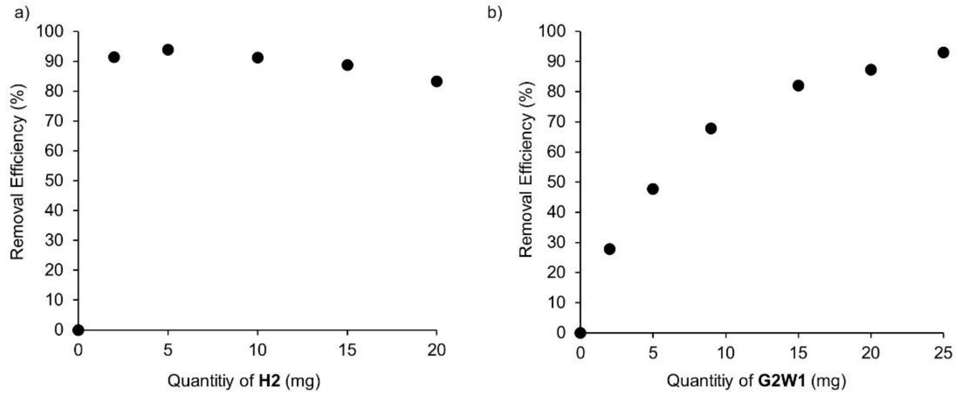


**Figure S15.** a) Plot of removal efficiency of methylene blue (240 μM, 1 mL) using different quantities of **H2** (2, 5, 10, 15, 20 mg). b) Plot of removal efficiency of methylene violet (38 μM, 6.4 mL) using different quantities of **G2W1** (2, 5, 10, 15, 20, 25 mg).

**Sequestration capability of H2 with different methylene blue concentrations.** **H2** (5.0 mg, 7.26 μmol) was incubated separately with different concentrations (1000, 300, 240, 180, 120, 90, 70 μM) of aq. methylene blue solutions (1 mL) separately for 1 h at 25 °C using a ThermoMixer™. The mixtures were centrifuged (11,000 rpm, 10 min), and the supernatants were analyzed by UV–vis spectroscopy. The removal efficiency was determined by using Equation S1.

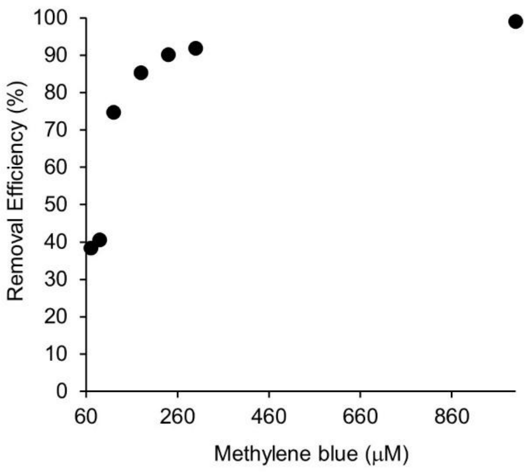


**Figure S16.** Sequestration capability of **H2** with different methylene blue concentrations.

**Rate of methylene blue and methylene violet uptake by H2 and G2W1, respectively.** The respective dyes ((methylene blue; 240 μM, 1 mL), (methylene violet; 38 μM, 6.4 mL)) were incubated with **H2** (5.0 mg) and **G2W1** (9.0 mg) respectively for different periods of time at 25 °C using a ThermoMixer™. For methylene blue with **H2** after each time interval (5, 10, 15, 30, 45, 60 min), the mixtures were centrifuged (11,000 rpm, 10 min), and the supernatants were analyzed by UV–vis spectroscopy. For methylene violet with **G2W1** after each time interval (1, 2, 5, 10, 30, 60 min), the mixtures were centrifuged (7500 rpm, 1 min), and the supernatants were analyzed by UV–vis spectroscopy. The removal efficiency was determined by using Equation S1.

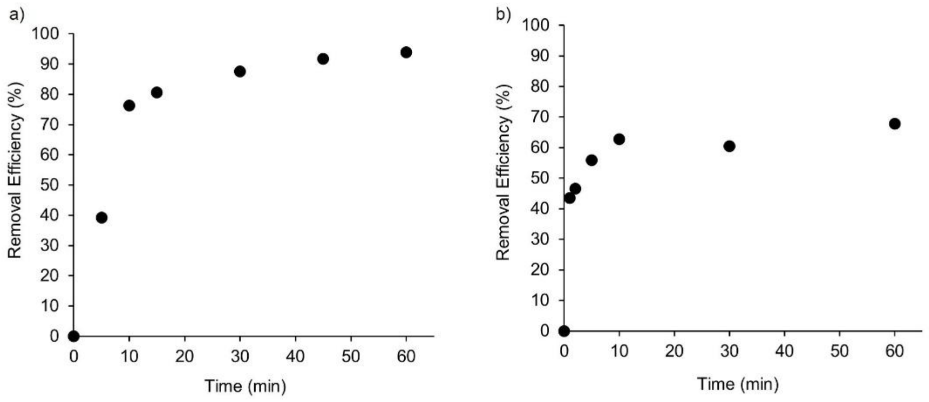


**Figure S17.** Removal efficiency at different time intervals: a) methylene blue by **H2**, b) methylene violet by **G2W1**.

**Langmuir isotherm for methylene violet.** Langmuir adsorption isotherm for methylene violet was generated by fitting the data into Equation S2 where $q_e$ (mg g$^{-1}$) is the amount of methylene violet adsorbed at equilibrium, $c$ (mol L$^{-1}$) is the residual methylene violet concentration at equilibrium, and $K$ (mol$^{-1}$) is the equilibrium constant.

$$\frac{1}{q_e} = \frac{1}{q_{max,e}} + \frac{1}{q_{max,e}.K.C} \qquad \text{(S2)}$$

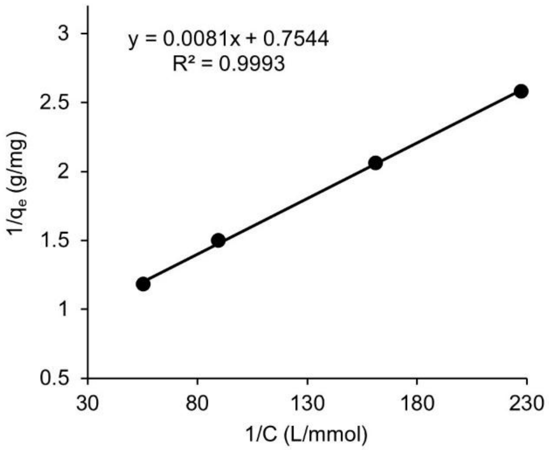


**Figure S18.** The Langmuir adsorption isotherm for the adsorption of methylene violet by **G2W1**.

**Crystallization of G2W1 and G2W3.** Crystals of **G2W1** and **G2W3** were generated by standard vapor diffusion methods. For **G2W1**: a solution of **G2W1** (10 mg) in $CHCl_3$ (1 mL) in a small uncapped vial was placed into a large jar containing MeOH as antisolvent and then the larger jar was capped. The jar was left at room temperature in the dark for several days during which single crystals formed. For **G2W3**: a solution of **G2W3** (10 mg) in TFA (1 mL) in a small uncapped vial was placed into a large jar containing MeOH as antisolvent and then the larger jar was capped. The jar was left at room temperature in the dark for several days during which single crystals formed.

## References

[1] Dorel, R.; Manzano, C.; Grisolia, M.; Soe, W.-H.; Joachim, C.; Echavarren, A. M. *Chem. Commun.* **2015**, *51*, 6932-6935.
[2] Maddala, S.; Panua, A.; Venkatakrishnan, P. *Chem. Eur. J.* **2021**, *27*, 16013-16020.
[3] Perera, S.; Shaurya, A.; Baptiste, M.; Zavalij, P. Y.; Isaacs, L. *Angew. Chem. Int. Ed.* **2024**, e202407169.
[4] Miyamura, H.; Tobita, F.; Suzuki, A.; Kobayashi, S. *Angew. Chem. Int. Ed.* **2019**, *58*, 9220-9224.
